# Activation of Molecular Oxygen by Electron‐Rich Materials for Sustainable Soil Remediation

**DOI:** 10.1002/advs.202518117

**Published:** 2025-12-01

**Authors:** Hengxin Liu, Yueming Han, Ruohan Li, Yuntao Guan, Lixun Zhang

**Affiliations:** ^1^ Guangdong Provincial Engineering Technology Research Center for Urban Water Cycle and Water Environment Safety Institute of Environment and Ecology Shenzhen International Graduate School Tsinghua University Shenzhen 518055 P. R. China

**Keywords:** electron‐rich materials, influencing factors, modification strategy, molecular oxygen activation, soil remediation

## Abstract

Traditional advanced oxidation processes (AOPs) in soil remediation rely on external chemical oxidants, which have evident drawbacks of high cost, soil structure disruption, and high carbon footprint. Compared to traditional AOPs, molecular oxygen (O_2_) activation requires no external reagents, and can realize environment‐friendly oxidation with inexhaustible O_2_ resources. Electron‐rich materials can activate O_2_ to generate reactive oxygen species (ROS) to achieve in situ remediation of soil pollution. However, there is lack of knowledge about O_2_‐mediated oxygenation reactions for pollutant removal in soil. Therefore, this review systematically investigated the common electron‐rich materials that can be used for O_2_ activation in soil, including iron‐based materials, bismuth‐based materials, copper‐based materials, and porous carbon. The mechanisms of O_2_ activation by these materials are summarized. Multiple methods, including vacancy defect, heteroatom doping, and edge defect can be applied to modify these materials for improving their electron transfer capacities toward O_2_ activation. The applications of electron‐rich materials‐driven O_2_ activation in soil remediation are discussed, and pH, oxygen content, organic matter, and sunlight intensity are identified as main influencing factors on ROS generation for pollutant removal. The outcomes will provide important implications for the development of O_2_‐mediated oxygenation technology to achieve sustainable and efficient soil remediation.

## Graphical abstract

Abundant molecular oxygen (O_2_) in nature can be utilized and activated by electron‐rich materials to generate reactive oxygen species for in situ engineering remediation of contaminated soil. The material types, activation mechanisms, and influencing factors are discussed in the research. This work will provide important implications for driving O_2_‐mediated oxygenation reactions for soil green and sustainable remediation.

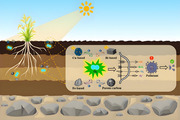

## 1 Introduction

With the rapid development of industry and agriculture, soil pollution has become a global environmental problem. Organic pollutants such as polycyclic aromatic hydrocarbons (PAHs), halogenated organic compounds, and pesticides enter the soil through industrial discharges and accidental spills, leading to degradation of ecosystem functions and threatening human health^[^ ^1^, ^2^, ^3^ ^]^ It is crucial to develop soil remediation techniques for efficient removal of pollutants from soil.^[^ ^4^, ^5^, ^6^, ^7^, ^8^ ^]^

Advanced oxidation processes (AOPs) have raised much attentions in soil remediation because of their fast reaction rate, thorough mineralization, and wide applicability.^[^ ^9^, ^10^, ^11^ ^]^ AOPs can achieve an efficient degradation of pollutants by generating reactive oxygen species (ROS). However, despite the remarkable efficacy of these technologies, the practical application of many AOPs in soil remediation remains constrained by inherent challenges. Fenton, Fenton‐like, and persulfate systems require substantial doses of chemical oxidants, and tend to disrupt soil environments and cause secondary pollution.^[^ ^12^, ^13^, ^14^ ^]^ Although electrochemical oxidation offers high controllability, it needs a long‐term external power supply and is limited by restricted contact between the electrode and the soil.^[^ ^15^, ^16^ ^]^ Photocatalysis in soil applications is constrained by light penetration rates, and ROS generation is restricted to the topsoil layer with deep soil remediation remaining unattainable.^[^ ^17^, ^18^ ^]^ Ozone struggles to permeate uniformly within soil pores and is readily decomposed non‐selectively by soil minerals and organic matter, which limits the large‐scale application of ozone oxidation technology in soil remediation.^[^ ^19^, ^20^ ^]^ The energy utilization efficiency of ultrasonic oxidation is relatively low. Soil particles scatter and absorb ultrasonic waves, and the energy attenuates sharply with distance.^[^ ^21^, ^22^ ^]^ Besides, plasma oxidation consumes high energy and incurs substantial equipment and operational costs. Moreover, its limited range of action makes it difficult to achieve deep‐seated in‐situ remediation of soil.^[^ ^23^, ^24^ ^]^ Therefore, the development of approaches featuring low chemical reagent addition, low energy consumption, and excellent mass transfer performance has become the key to upgrade AOPs technology in the field of soil remediation.

The activation and utilization of molecular oxygen (O_2_) as a cheap and easily available oxidant precursor provides a new direction for the sustainability of AOPs. In contrast to conventional AOPs, O_2_ activation represents a greener and low‐carbon alternative. O_2_ is readily available and environmentally benign, and requires no external reagent injection to avoid soil structure disruption. Moreover, O_2_ can be continuously supplied to the soil through plant photosynthesis and root oxygen exudation.^[^ ^25^, ^26^ ^]^ Electron‐rich materials can drive electron transfer at the interfacial scale to activate O_2_. The generation of ROS, such as superoxide radicals ($O_{2}^{\cdot -}$), hydrogen peroxide (H_2_O_2_), and hydroxyl radicals (·OH), can achieve in situ remediation of soil contamination.^[^ ^27^, ^28^, ^29^ ^]^ This system features a low carbon footprint and can operate under low‐light or even dark conditions. Furthermore, it enables a continuous supply of electrons for electron‐rich materials via slow oxidation of organic matter, extracellular electrons generated by microbial metabolism, seasonal wet‐dry cycles, and tidal fluctuations.^[^ ^30^, ^31^, ^32^ ^]^ Studies showed that the system could remove 36%–80% of organic pollutants like naphthalene, carbamazepine, and bisphenol A within hours to days.^[^ ^33^, ^34^, ^35^, ^36^ ^]^ Electron‐rich materials refer to substances with the capacity to donate reactive electrons to O_2_ and drive interfacial electron transfer. They exhibit a work function below 4.5 eV and a Fermi level density of states exceeding 1.0 states/eV/atom.^[^ ^37^, ^38^, ^39^, ^40^ ^]^ Current research focuses on the development of transition metal catalysts and semiconductor photocatalysts, but these materials still face the problems of easy passivation of active sites and narrow light response range.^[^ ^41^, ^42^, ^43^, ^44^ ^]^ In recent years, some cheap and electron‐rich materials represented by zero‐valent iron (ZVI), FeS, and biochar have attracted much attentions due to their strong electron supplying ability and multi‐path activation mechanisms for O_2_.^[^ ^45^, ^46^, ^47^, ^48^ ^]^ For example, ZVI can activate O_2_ to generate ·OH through direct surface electron transfer as well as corrosion release of Fe^2+^, while its porous structure can promote the interfacial reaction between pollutants and ROS.^[^ ^49^, ^50^, ^51^ ^]^ FeS can promote the successive reduction of O_2_ to generate various ROS by releasing reactive electrons through the oxidation of surface Fe(II). Its surface sulfur (S) species can act as an electron transfer medium to accelerate the Fe(II)/Fe(III) cycle and stabilize the intermediates to enhance O_2_ activation efficiency.^[^ ^52^, ^53^ ^]^ In addition, biochar facilitates the electron transfer process of O_2_ through its abundant surface functional groups and conductive carbon skeleton. Its porous structure not only enhances the pollutant adsorption capacity, but also facilitates the activation of O_2_ to generate $O_{2}^{\cdot -}$ and single‐linear oxygen (^1^O_2_) through defect sites and edge carbon atoms.^[^ ^54^, ^55^ ^]^

Current reviews on O_2_ activation primarily concentrate on O_2_ activation methods, ROS generation mechanisms, and remediation in water and air pollution.^[^ ^56^, ^57^, ^58^ ^]^ However, the evolution patterns of electron‐rich materials for O_2_ activation remain unclear in complex soil matrices with organic matter and clay minerals. Quantitative analyses of synergistic or antagonistic effects of different environmental factors on ROS generation are lacking. Furthermore, the interfacial mechanisms of multi‐material synergistic O_2_ activation need further clarification. Considering the abundant O_2_ resource in natural environments, it is a promising and sustainable strategy to reduce them to produce ROS by electron‐rich materials for soil remediation. Compared to previous reviews, this paper provides the first systematic summary of the mechanism of electron‐rich materials driving O_2_ activation and its modification methods for enhancing O_2_ activation. Furthermore, the applications and influencing factors of electron‐rich materials for soil pollution remediation via activating O_2_ were investigated. This work aims to provide a theoretical support and strategic guidance for the development of low‐consumption and high‐efficiency soil remediation technology.

## 2 Types and Mechanisms of Electron‐Rich Materials for the Activation of O_2_

**Figure 1:**
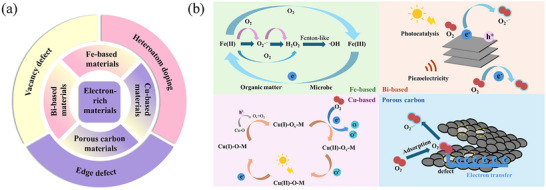
a) Classification of electron‐rich materials for O_2_ activation and common modification methods. b) Mechanisms of O_2_ activation by different types of electron‐rich materials.

Based on their comprehensive advantages in electronic structure, reactivity, and environmental applicability, the mechanisms of O_2_ activation by four categories of electron‐rich materials were summarized. As shown in **Figure** **1**a, electron‐rich materials for O_2_ activation can be mainly divided into iron (Fe)‐based materials, bismuth (Bi)‐based materials, copper (Cu)‐based materials, and porous carbon materials. As indicated in Figure 1b, Fe‐based materials are the most common transition metal minerals in soil and possess abundant sources and environmental friendliness. With reversible Fe(II)/Fe(III) redox cycles, it can efficiently drive multi‐electron reduction of O_2_ to generate ROS. Next, Bi‐based materials have unique layered structures and tunable band positions, and combine light responsiveness with environmental stability. It can realise efficient electron‐hole separation under light or piezoelectric action and thus promote O_2_ activation. Moreover, Cu‐based materials can promote O_2_ adsorption and activation due to rapid electron migration between Cu^+^ and Cu^2+^ states and excellent catalytic tunability. Finally, porous carbon materials with high specific surface area, superior conductivity, and controllable defect structures serve as electron transfer frameworks to stabilize and enhance O_2_ activation at their surfaces.

### 2.1 Fe‐Based Materials

ZVI can effectively activate O_2_ to produce $O_{2}^{\cdot -}$, H_2_O_2_, and ·OH through its strong reducibility and surface reactivity. Usually, the ZVI‐induced O_2_ activation process occurs as follows. First, Fe^0^ provides two electrons to O_2_ to reduce it to H_2_O_2_ while Fe^0^ is oxidized to Fe(II) (Equation (1)). Next, Fe(II) activates O_2_ by single electron transfer to produce $O_{2}^{\cdot -}$ (Equation (2)), which can further react with Fe(II) to produce H_2_O_2_ (Equation (3)). Eventually, the generated H_2_O_2_ can react with Fe(II) to produce ·OH at pH 3–5 (Equation (4)), which can be applied to pollutant degradation.^[^ ^40^, ^59^, ^60^ ^]^

(1) $$O_{2}+\;Fe^{0}+2H^{+}\;\to \;\mathrm{Fe}\left(\mathrm{II}\right)+H_{2}O_{2}$$

(2) $$O_{2}+\mathrm{Fe}\left(\mathrm{II}\right)\to \mathrm{Fe}\left(\mathrm{III}\right)+O_{2}^{\cdot -}$$

(3) $$O_{2}^{\cdot -}+\;\mathrm{Fe}\left(\mathrm{II}\right)+2H^{+}\to \mathrm{Fe}\left(\mathrm{III}\right)+H_{2}O_{2}$$

(4) $$H_{2}O_{2}+\mathrm{Fe}\left(\mathrm{II}\right)\;\to \mathrm{Fe}\left(\mathrm{III}\right)+\cdot \mathrm{OH}+{\mathrm{OH}}^{-}$$

FeS is a common form of iron sulfide in soil. Due to its structural instability and strong reducing ability, it easily reacts with O_2_ to produce ROS to degrade pollutants. In a neutral environment, O_2_ is mainly activated by structural Fe(II) of FeS via a heterogeneous reaction, which first undergoes a two‐electron transfer to generate H_2_O_2_ intermediates (Equation (5)). Next, H_2_O_2_ and Fe(II) interact in a Fenton reaction to produce ·OH (Equation (6)). In addition to structuring Fe(II), S can act as an electron donor and mediate the Fe cycle. The non‐oxidative dissolution of FeS occurs to release Fe^2+^ and H_2_S under pH 4–6, and ·OH is further generated in the homogeneous Fenton reaction catalyzed by the dissolved Fe^2+^ (Equations (7) and (8)).^[^ ^61^, ^62^, ^63^ ^]^

(5) $$\equiv \mathrm{Fe}\left(\mathrm{II}\right)+O_{2}+2H^{+}\to \mathrm{Fe}\left(\mathrm{III}\right)+H_{2}O_{2}$$

(6) $$H_{2}O_{2}+\mathrm{Fe}\left(\mathrm{II}\right)\to \equiv \mathrm{Fe}\left(\mathrm{III}\right)+\cdot \mathrm{OH}+OH^{-}$$

(7) $$\mathrm{FeS}+2H^{+}\;\to \;\mathrm{Fe}{\left(\mathrm{II}\right)}_{\left(\mathrm{aq}\right)}+H_{2}S_{\left(\mathrm{aq}\right)}$$

(8) $$\mathrm{Fe}{\left(\mathrm{II}\right)}_{\left(\mathrm{aq}\right)}+H_{2}O_{2}\to \mathrm{Fe}{\left(\mathrm{III}\right)}_{\left(\mathrm{aq}\right)}+\cdot \mathrm{OH}+{\mathrm{OH}}^{-}$$

In contrast to FeS, FeS_2_ is more stably distributed in soil. The O_2_ activation by FeS_2_ is mainly dependent on surface Fe(II) and S vacancies. Structural Fe(II) and surface‐bound Fe(II) can activate O_2_ via a two‐electron transfer (Equation (5)) and two independent one‐electron transfer processes (Equations (9) and (10)), respectively, and the generated H_2_O_2_ and $O_{2}^{\cdot -}$ intermediates can be converted to ·OH. Moreover, with the low‐rate solubilization of sulfide iron ore, a small portion of dissolved Fe(II) can activate O_2_ via a one‐electron transfer pathway (Equations (11)–(13)). In particular, S vacancies play a key role in the O_2_ activation process. Initially, surface S vacancies can be hydrolyzed directly to generate ·OH, and then react with $O_{2}^{\cdot -}$ to form ^1^O_2_ (Equations (14) and (15)). Also, Fe(III) oxides on the surface of FeS_2_ containing S vacancies can promote the conversion of O_2_ to H_2_O_2_, and further to ^1^O_2_ (Equations (16) and (17)).^[^ ^64^, ^65^, ^66^, ^67^, ^68^ ^]^ Overall, FeS exhibits stronger electron supply and reduction capabilities due to its higher proportion of Fe(II), and is suitable for the rapid initiation of O_2_ reduction in weakly reducing or low‐oxygen environments. In contrast, FeS_2_ possesses a stable ligand structure with band positions more conducive to electron transfer, and shows higher efficacy in neutral or oxidative environments.

(9) $$\mathrm{Fe}{\left(\mathrm{II}\right)}_{\mathrm{ad}}+O_{2}\;\to \mathrm{Fe}\left(\mathrm{III}\right)+O_{2}^{\cdot -}$$

(10) $$O_{2}^{\cdot -}+\mathrm{Fe}\left(\mathrm{II}\right)+2H^{+}\;\to \mathrm{Fe}\left(\mathrm{III}\right)+H_{2}O_{2}$$

(11) $$\mathrm{Fe}{\left(\mathrm{II}\right)}_{\left(\mathrm{aq}\right)}+O_{2}\to \mathrm{Fe}{\left(\mathrm{III}\right)}_{\left(\mathrm{aq}\right)}+O_{2}^{\cdot -}$$

(12) $$\mathrm{Fe}{\left(\mathrm{II}\right)}_{\left(\mathrm{aq}\right)}+O_{2}^{\cdot -}+2H^{+}\to \mathrm{Fe}{\left(\mathrm{III}\right)}_{\left(\mathrm{aq}\right)}+H_{2}O_{2}$$

(13) $$\mathrm{Fe}\left(\mathrm{II}\right)+H_{2}O_{2}\to \mathrm{Fe}\left(\mathrm{III}\right)+\cdot \mathrm{OH}+OH^{-}$$

(14) $$\mathrm{Fe}{\left(\mathrm{III}\right)}_{\left(\mathrm{SV}-\mathrm{Pyrite}\right)}+H_{2}O\to \mathrm{Fe}{\left(\mathrm{II}\right)}_{\left(\mathrm{SV}-\mathrm{Pyrite}\right)}+\cdot OH_{\left(\mathrm{ad}\right)}+H^{+}$$

(15)

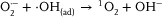

(16) $$\mathrm{Fe}{\left(\mathrm{III}\right)}_{\left(\mathrm{SV}-\mathrm{Pyrite}\right)}+O_{2}\;\to H_{2}O_{2}$$

(17)

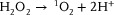




The major Fe oxides in soil include magnetite (Fe_3_O_4_), hematite (α‐Fe_2_O_3_), and clinopyroxene (α‐FeOOH). Due to the presence of Fe(II) in Fe_3_O_4_, O_2_ can be activated by single electron transfer to generate $O_{2}^{\cdot -}$, and then $O_{2}^{\cdot -}$ is converted to H_2_O_2_ and ·OH with a chain reaction. Fang et al. investigated the O_2_ activation properties of Fe_3_O_4_ nanoparticles. Results indicated that Fe_3_O_4_ possessed a specific surface area of 83.5 m^2^g^−1^, with Fe(II) in the surface or lattice serving as the key site for ROS generation. The Fe(II)/Fe(III) ratio was ≈0.43 before the reaction and decreased to 0.27 after the reaction. The removal rate of 2‐chlorobiphenyl (2 mgL^−1^) reached ≈80% within 180 min.^[^ ^69^ ^]^ Furthermore, the addition of suitable ligands such as trimeric phosphates, cysteines, and pyrophosphates can lead to the formation of Fe(II) complexes. It can facilitate the stretching of Fe─O bonds and expose more Fe active sites, consequently, the O_2_ activation efficiency is enhanced.^[^ ^70^, ^71^, ^72^ ^]^ However, the Fe speciation in both α‐Fe_2_O_3_ and α‐FeOOH exists as Fe(III), and it is difficult for them to reduce O_2_ directly. Therefore, they need to rely on external reducing agents to provide electrons for the reduction of Fe(III) to Fe(II) to activate O_2_.^[^ ^40^ ^]^

α‐Fe_2_O_3_ with a bandgap of about 2.1–2.2 eV can generate electrons (e^−^) and holes (h^+^) under visible light excitation on the soil surface. The photogenerated electrons can migrate to the conduction band and reduce the adsorbed O_2_ molecules to generate $O_{2}^{\cdot -}$ through interfacial transfer. h^+^ can be involved in the oxidation of water or organic matter to generate ·OH.^[^ ^73^, ^74^, ^75^ ^]^ Su et al. demonstrated that α‐Fe_2_O_3_ exhibited limited efficacy in the removal of pollutants via photocatalysis. With a specific surface area of only 19.5 m^2^g^−1^, α‐Fe_2_O_3_ generated ·OH under photocatalytic conditions. However, its carrier lifetime was merely 2.7 ns, and the removal efficiency for tetracycline (20 mgL^−1^) reached only 20.6%.^[^ ^75^ ^]^ Dissolved organic matter on the surface of α‐Fe_2_O_3_ can also reduce Fe(III) to Fe(II) by electron transfer under natural light irradiation, and reduce O_2_ to $O_{2}^{\cdot -}$ by single electron transfer.^[^ ^76^ ^]^

α‐FeOOH on the soil surface under pH 5–6.5 can form ≡FeOH complexes on the surface by ligand‐metal charge transfer under photoexcitation, and facilitate the production of dissolved Fe(II) and ·OH (Equation (18)). Next, O_2_ adsorbed on the surface of α‐FeOOH can be reduced to H_2_O_2_ by two single‐electron transfers, and ·OH can be further generated through Fenton reaction.^[^ ^77^, ^78^ ^]^ Yuan et al. demonstrated that the ≡Fe‐OH groups on the α‐FeOOH surface were the key sites for adsorption and photocatalytic activity. With a specific surface area of 85.1 m^2^ g^−1^, α‐FeOOH could form complexes with roxarsone through ligand exchange. The maximum adsorption capacity reached 0.19 mmol g^−1^, approximately reflecting the density of active sites. The removal rate of rosazone (5 µmol/L) via photocatalysis at pH 3 was about 40% within 6 h.^[^ ^77^ ^]^

(18) $$\equiv \mathrm{FeOH}\;\overset{\mathrm{hv}}{\to }\;\mathrm{Fe}{\left(\mathrm{II}\right)}_{\left(\mathrm{aq}\right)}+\cdot \mathrm{OH}$$

Green rust (GR) is another common Fe‐based clay material. The mechanisms of O_2_ activation by GR are mainly based on the dynamic transformation of Fe(II) under oxidative conditions and the generation of ROS.^[^ ^79^, ^80^, ^81^ ^]^ GR can first be oxidized to Fe_3_O_4_ in aerobic environment. The adsorbed Fe(II) on its surface and free Fe(II) in solution can gradually reduce O_2_ to $O_{2}^{\cdot -}$, H_2_O_2_, and finally generate highly reactive ·OH.^[^ ^82^, ^83^ ^]^ Besides, the pathway of O_2_ activation via GR can be broadened by doping metals. For example, Cu(II) in the Cu(II)‐GR(II) system can be reduced to Cu(I) by Fe(II), and Cu(I) is involved in the O_2_ activation (Equations (19) and (20)).^[^ ^83^ ^]^ Similarly, in the Pd(II)‐Cu(II) system, Pd(II) is reduced to Pd(0) with Fe(II), and Pd(0) transforms H_2_O to atomic hydrogen (H^*^). Moreover, H_2_O· is generated by the reaction between H^*^ and O_2_ and is further transformed into H_2_O_2_ (Equations (21)–(24)).^[^ ^84^ ^]^ Notably, the interlayer anions in GR may play a key role in O_2_ activation.^[^ ^85^, ^86^, ^87^, ^88^ ^]^ Hydrophobic intercalation anions (e.g., C_12_H_25_SO_4_^−^ and C_18_H_33_O_2_^−^) can form hydrophobic environments in the interlayer and facilitate the O_2_ enrichment on the GR surface. Hence, the contact efficiency between O_2_ and Fe(II) active sites can be enhanced.^[^ ^86^, ^89^, ^90^ ^]^ Furthermore, anions can be stably retained in the interlayer due to the hydrophobic interactions of the alkyl chains. In this way, the layered structure of GR can be preserved in the oxidization process, and the conversion to Fe oxides, such as acicular ferrite or Fe_3_O_4_ is avoided. Consequently, the highly reactive surface essential for O_2_ activation is maintained.^[^ ^91^ ^]^

(19) $$\mathrm{Fe}\left(\mathrm{II}\right)+\mathrm{Cu}\left(\mathrm{II}\right)\to \mathrm{Fe}\left(\mathrm{III}\right)+\mathrm{Cu}\left(I\right)$$

(20) $$\mathrm{Cu}\left(I\right)+O_{2}+2H^{+}\to \mathrm{Cu}\left(\mathrm{II}\right)+H_{2}O_{2}$$

(21) $$\mathrm{Fe}\left(\mathrm{II}\right)+\mathrm{Pd}\left(\mathrm{II}\right)\to \mathrm{Fe}\left(\mathrm{III}\right)+\mathrm{Pd}\left(0\right)$$

(22) $$\mathrm{Pd}\left(0\right)+H_{2}O\to \mathrm{Pd}\left(\mathrm{II}\right)+H*+OH^{-}$$

(23) $$H*+O_{2}\;\to {\mathrm{HO}}_{2}^{\cdot }$$

(24) $${\mathrm{HO}}_{2}^{\cdot }+H^{+}\to H_{2}O_{2}$$

### 2.2 Bi‐Based Materials

Heterojunction composite structures can be formed by interfacial contact between Bi‐based semiconductors and other semiconductors. Synergistic modulation of energy band structures and efficient separation of interfacial charges are provided as features. The energy band discontinuity and the difference in spatial charge distribution on the heterojunction interface can generate an electric field to promote the separation of photogenerated electron‐hole pairs.^[^ ^92^, ^93^, ^94^ ^]^ Meanwhile, the high local charge density and optimized adsorption sites at the interface can enhance the chemical adsorption capacity of O_2_ and offer favorable conditions for O_2_ activation.^[^ ^95^, ^96^ ^]^ The mechanisms of O_2_ photocatalytic activation via Bi‐based semiconductor heterojunction materials can be divided into the following steps. Initially, e^−^ and h^+^ are produced by the electron jump from the valence band (VB) to the conduction band (CB) under photoexcitation. Owing to the energy band coordination of heterojunction, the e^−^ is transferred from one semiconductor's CB to another one's VB, and spatial segregation of carriers is realized. Moreover, O_2_ adsorbed on the interface is reduced to $O_{2}^{\cdot -}$ by photogenerated electrons. And it further reacts with h⁺ to form ^1^O_2_ or interacts with H_2_O to form ·OH. The actual pathway depends on the energy band position and reaction environment of the system.^[^ ^97^, ^98^ ^]^

Another type of Bi‐based materials is developed as piezoelectric materials for O_2_ activation through the interconversion of mechanical and electrical energy. With high curie temperature (>400 °C), low dielectric loss, and remarkable anisotropic piezoelectric coefficient, they show significant benefits in O_2_ activation applications. The activation mechanisms are mainly based on the synergistic interaction between the built‐in electric field induced by the piezoelectric effect and the surface active sites.^[^ ^99^, ^100^, ^101^ ^]^ As external mechanical stress is applied to the Bi‐based piezoelectric material, the lattice distortion causes the separation of positive and negative charge centers to form a piezoelectric field. This drives the migration of free electrons toward the material surface.^[^ ^102^ ^]^ Simultaneously, the Bi oxygen layer or doping sites on the material surface can be used as active centers to adsorb O_2_ and generate $O_{2}^{\cdot -}$ with electron transfer. Furthermore, e^−^ can drive the dynamic redox cycle of the active sites on doped metals (e.g., Fe) as well as the cascade radical reaction, and realize the efficient O_2_ activation and the deep degradation of organic pollutants.^[^ ^103^ ^]^

### 2.3 Cu‐Based Materials

Cu‐based monoatomic catalysts are atomically dispersed Cu anchored on the surface of a metal carrier. They form a unique Cu‐O‐M coordination structure by the substitution of some lattice atoms (M is the metal substrate atom). The material with high atomic utilization can synergistically activate O_2_ through Cu active centers and adjacent lattice oxygen sites, and active sites can be dynamically reconfigured by photoinduction.^[^ ^104^, ^105^, ^106^ ^]^ O_2_ activation in Cu‐based single‐atom materials is realized with the following synergistic effects. Initially, the Cu(II)‐O‐M coordination structure is generated via atomically dispersed Cu sites and carriers. Under photogenerated electron transfer, Cu(II) is reduced to Cu(I) (Equation (25)) to produce Cu(I)‐O‐M. Also, photogenerated O_v_ can be formed in this process to create Cu(I)‐O_v_‐M active sites, and it is favorable for the O_2_ adsorption on O_v_ sites in a terminated configuration. In this case, O_2_ is activated by Cu(I) through electron transfer to generate monatomic oxygen ions O^2−^ and O^−^ (Equation (26)), and Cu(I) is converted into Cu(II). Furthermore, O^−^ can participate in the oxidative degradation of pollutants, and O^2−^ can fill the photogenerated O_v_ for the Cu(II)‐O‐M regeneration. Hence, the redox cycle is continuously performed in Cu monoatomic centers by the combined action of photogenerated electrons and O_2._^[^ ^107^, ^108^ ^]^ Although Cu‐based monoatomic catalysts have demonstrated outstanding O_2_ activation performance and high atomic utilization in research, their application in actual soil environments faces certain limitations. Monoatomic structures are prone to agglomeration and deactivation within complex heterogeneous systems, while the environmental stability of support materials may be affected by fluctuations in soil pH, organic matter content, and moisture levels. Furthermore, cations, anions, natural organic matter, and microorganisms may compete for adsorption or form complexes with active sites, and thus diminish catalytic efficiency. Therefore, further development of Cu‐based monoatomic catalysts with high stability and resistance to interference is required.

(25) $$\mathrm{Cu}\left(\mathrm{II}\right)+e^{-}\to \mathrm{Cu}\left(I\right)$$

(26) $$\mathrm{Cu}\left(I\right)+O_{2}\;\to \mathrm{Cu}\left(\mathrm{II}\right)+O^{2^{-}}+O^{-}$$

Cu‐based metal‐organic skeletons (Cu‐MOFs) are porous crystalline materials with high specific surface area, tunable pore size structure, and abundant metal active sites. Cu‐MOFs are formed by self‐assembly between Cu ions or Cu clusters as metal nodes and organic ligands via ligand bonding. In general, Cu(II)‐MOFs are usually activated through charge transfer in photocatalytic or electrochemical systems to activate O_2._^[^ ^109^, ^110^, ^111^ ^]^ For Cu(0)‐MOFs, the Cu(0) on the surface of the Cu‐MOFs lose their electrons and are oxidized to Cu(I) and Cu(II). The released electrons prompted a two‐electron reduction of O_2_ to form H_2_O_2_ (Equations ((27), (28), (29))). Then, H_2_O_2_ can generate Cu(III) and ·OH catalyzed by Cu(I) sites (Equations (30) and (31)), and further facilitate the oxidative decomposition of pollutants.^[^ ^112^, ^113^ ^]^ Besides, some Cu‐MOFs synergistically break the O─O bond by the construction of dinuclear Cu sites (e.g., Cu_2_O_2_ clusters) to create ROS, and enhance the O_2_ activation efficiency.

(27) $$\mathrm{Cu}\left(0\right)\;-e^{-}\;\to \;\mathrm{Cu}\left(I\right)$$

(28) $$\mathrm{Cu}\left(I\right)-e^{-}\;\to \mathrm{Cu}\left(\mathrm{II}\right)$$

(29) $$O_{2}+2e^{-}+2H^{+}\to H_{2}O_{2}$$

(30) $$\mathrm{Cu}\left(I\right)+H_{2}O_{2}\to \mathrm{Cu}\left(\mathrm{II}\right)+\cdot \mathrm{OH}+2OH^{-}$$

(31) $$\mathrm{Cu}\left(I\right)+H_{2}O_{2}+2H^{+}\to \mathrm{Cu}\left(\mathrm{III}\right)+2H_{2}O$$

### 2.4 Porous Carbon Materials

Porous carbon materials, such as biochar, are carbon‐based materials with a network structure consisting of interconnected or closed pores. Their pore size can be categorized into micropores (<2 nm), mesopores (2–50 nm), and macropores (>50 nm) according to the International Union of Pure and Applied Chemistry standards. Such materials have high specific surface area, well‐developed pore structure, excellent electrical conductivity, and chemical stability. The domain‐limiting effect of their pores and surface electronic property shows unique advantages for O_2_ activation.^[^ ^114^, ^115^, ^116^ ^]^ The entry of O_2_ molecules into microporous or mesoporous structures imposes spatial constraints on their adsorption configurations, and reduces the stretching vibrational degrees of freedom of the O─O bond. This alters molecular orbital energy levels and enhances the affinity of the π^*^ orbital for electrons. Concurrently, defect states and edge states on the pore walls induce localized electron density enrichment, enhance the polarization of O_2_, and facilitate interfacial electron transfer^[^ ^117^, ^118^, ^119^ ^]^ Mesopores and macropores can be employed as transport channels to accelerate the reactant diffusion. Moreover, it was shown that the domain‐limited space of ultra‐micropores (≈0.4 nm) can significantly enhance the interaction between O_2_ and carbon skeleton. The sp^2^ hybridized structure of the carbon material transfers electrons to the antibonding tracks of O_2_ via π^*^ orbitals, and O_2_ acquires a single electron for $O_{2}^{\cdot -}$ formation (**Equation (32**)).^[^ ^117^, ^120^ ^]^

(32) $$O_{2}+e^{-}\to O_{2}^{\cdot -}$$

Overall, as shown in **Table** **1**, Fe‐based materials rely on Fe(II)/Fe(III) cycle‐driven O_2_ reduction and exhibit stability under weakly acidic to neutral conditions. Bi‐based materials possess visible‐light responsiveness advantages but show strong light dependence. Cu‐based materials demonstrate high electron transfer efficiency yet exhibit limited stability in complex ionic environments. Porous carbon materials, with their excellent conductivity and interfacial structure, can achieve sustained O_2_ activation without external oxidants. Notably, the degradation pathways and final toxicity changes of pollutants exhibit significant differences across various O_2_‐activated systems. In systems dominated by ·OH on Fe‐based and Cu‐based materials, pollutants typically undergo non‐selective hydroxylation, ring‐opening, and chain oxidation processes with higher mineralization rates. Conversely, systems dominated by $O_{2}^{\cdot -}$ or ^1^O_2_ on Bi‐based and porous carbon materials exhibit reactions that tend toward oxygenation, dehydrogenation, substitution, and partial bond‐breaking pathways with lower toxicity than the original pollutants.

**Table 1:** Comparison of similarities and differences among electron‐rich materials.

Material type	Main modification strategy	Activation mechanism	Main ROS	Advantage	Limitation	Typical application condition	Refs.
Fe‐based materials	Vacancy defect	Fe(II)/Fe(III) redox cycle to drive O_2_ reduction	·OH	Low cost, abundant, suitable for in situ application	Sensitive to pH and O_2_ diffusion	Weakly acidic soils	[121, 122, 123]
Bi‐based materials	Heteroatom doping	Photoinduced electron reduction of O_2_	$O_{2}^{\cdot -}$, ^1^O_2_	Visible‐light responsive, tunable structure	Light‐dependent, prone to aggregation	Surface soils under illumination	[124, 125, 126]
Cu‐based materials	Vacancy defects and heteroatom doping	Cu(I)/Cu(II) redox cycle facilitating electron transfer	·OH	High electron mobility, adjustable active sites	Limited stability, ion interference	Neutral to mildly acidic soils	[127, 128, 129, 130]
Porous carbon materials	Edge defect and heteroatom doping	π‐electron donation and interfacial O_2_ adsorption activation	$O_{2}^{\cdot -}$, ^1^O_2_	Excellent conductivity, eco‐friendly	Nonuniform active sites, limited regeneration	Neutral or weakly oxidative soils	[131, 132, 133]

## 3 Modification Methods of Electron‐Rich Materials for the Improvement of O2 Activation

### 3.1 Vacancy Defect

The metal materials can be modified to a metal atomic vacancies or oxygen vacancies (OVs) structure to modulate their electronic properties for O_2_ activation.^[^ ^134^, ^135^ ^]^ The metal materials with vacancy defects can enhance the O_2_ activation from multiple pathways. Primarily, as electron‐rich centers, surface OVs can absorb O_2_ directly, and electrons are injected into antibonding orbitals of O_2_ to form $O_{2}^{\cdot -}$. Furthermore, the localized electric field induced by the vacancy can drive the rapid separation of photogenerated electron‐hole pairs. In this case, the electrons are migrated to surface OVs and further activate the adsorbed state O_2_. Besides, Metal atomic vacancy can increase the number of unpaired electrons via modulation of spin‐polarization behavior, and further reinforce the O_2_ adsorption and electron transfer.^[^ ^92^, ^103^ ^]^ Vacancy defects can be introduced into metallic materials through chemical reduction, high‐temperature calcination, and hydrothermal methods. Chemical reduction removes anions from crystals selectively via reductants and creates atomic vacancy defects in the crystal lattice. For example, Fe_2_O_3_ treated with KBH_4_ for 1 h showed a significant increase in OVs concentration and the removal rate of carbaryl increased by 74% within 30 min.^[^ ^122^ ^]^ Wang et al. synthesized Bi vacancy‐modified Bi_2_O_2_S through the reduction of Bi(NO_3_)_3_·5H_2_O and thioacetamide with NaBH_4_ for 12 h, which promoted the charge separation and O_2_ activation. As the mass ratio of NaBH_4_ to Bi(NO_3_)_3_ increased from 0 to 0.0808, the removal rate constant for Rhodamine B increased by 20.8 × 10^−3^ min^−1^. However, with a further increase to 0.1687, it decreased by 12.5 × 10^−3^ min^−1^. Therefore, the vacancy concentration should not be excessively high, as this may lead to carrier recombination and reduce O_2_ activation efficiency.^[^ ^136^ ^]^ Chemical reduction methods involve mild reaction conditions and provide controllable defect density and material structure, but the vacancy distribution is uneven, and it is difficult to reduce highly stable oxides. High‐temperature calcination refers to the treatment of materials at high temperatures (>350 °C) in an inert or reducing atmosphere to induce vacancy defects through atomic volatilization or phase transformation. Fe_2_O_3_@CeO_2_ heterojunctions introduced abundant OVs on the catalyst surface through calcination at 350 °C. The introduction of OVs optimized the electronic structure, reduced the O_2_ desorption energy barrier, and promoted the participation of lattice oxygen in the reaction.^[^ ^121^ ^]^ Also, Bi_5_Nb_3_O_15_ was calcined at 550 °C for 2 h. The Bi─O bonds broke to form OVs, and this promoted O_2_ adsorption and activation. The degradation rate constant for 2,4‐dichlorophenoxy acetic acid (2,4‐D) reached 1.3 × 10^−2^ min^−1^, and was 13.6 times higher than that of the original Bi_5_Nb_3_O_15_.^[^ ^137^ ^]^ Besides, Jiang et al. obtained Ovs‐rich Cu_2_O_1‐x_ nanoparticles through a 2 h calcination at 400 °C in a vacuum environment. OVs enhanced O_2_ activation to $O_{2}^{\cdot -}$ through improved conductivity, and reaction rate constant for perchloric acid removal increased from 2.6 × 10^−3^ min^−1^ to 2.3 × 10^−3^ min^−1^.^[^ ^128^ ^]^ The high‐temperature calcination process is simple with high defect concentration but requires considerable energy consumption and may cause particle agglomeration. The hydrothermal process refers to the formation of defect‐rich nanomaterials through the control of precursor crystallization under high temperature and pressure in a closed reactor. Zhang et al. introduced OVs into Fe_2_O_3_ in a stainless steel reactor at 160 °C for 24 h with ethylene glycol (EG), and the photocurrent density increased by 2.7 mAcm^−2^.^[^ ^123^ ^]^ In addition, Jin et al. dissolved Bi(NO_3_)_3_·5H_2_O and KI in EG and made them react at 160 °C for 16 h to obtain OVs‐rich Bi_4_O_5_I_2_. The results showed that the number of electrons transferred to O_2_ on the OVs surface (0.337 e) was significantly higher than that in the absence of vacancies (0.177 e). Moreover, the removal rate of nitroblue tetrazolium increased by ≈23% with the introduction of vacancies.^[^ ^138^ ^]^ Other researches showed CuS nanomaterials with Cu vacancies were formed through the reaction of CuCl_2_ and thiourea in 140 °C water solution for 8 h. Cu vacancies could extend the carrier lifetime and promote O_2_ activation as electron capture centers. The removal rate of methylene blue could reach 97% after 100 min of photocatalysis.^[^ ^127^ ^]^ Zhang et al. synthesized BiOBr rich in oxygen vacancies via hydrothermal synthesis at 160 °C for 12 h and controlled vacancy content by calcination in oxygen. The initial BiOBr‐O_V_ exhibited a 23.5% oxygen vacancy fraction in the XPS O 1s peak area and achieved an 88.9% removal rate for tetracycline within 120 min. With the increase in calcination temperature from 350 °C to 400 °C, the oxygen vacancy fraction decreased from 23.6% to 0%, while the tetracycline removal rate dropped from 80.1% to 73.4%.^[^ ^135^ ^]^ The materials synthesized by hydrothermal processes exhibit evenly distributed defects and diverse morphologies, and can form structures such as nanosheets, rods, and flowers. However, the reaction time is relatively long, and energy consumption is high.

Vacancy defects can cause local redistribution of electron density in porous carbon, and form regions of high spin density and unpaired electrons. As donors, these delocalized π electrons directly attack the π antibonding orbitals of O_2_, significantly weaken the strength of O─O bonds, and bypass the traditional ^*^OOH intermediate to achieve a dissociation pathway of direct O‐O bond cleavage. Besides, the porous structure can enhance the defect site density and local O_2_ concentration via the synergistic effects of high specific surface area and confinement effect. This consequently improves electron transfer efficiency. High‐temperature pyrolysis is usually an important step in the construction of vacancy defects in porous carbon. According to different pretreatment or posttreatment ways, vacancy defects in porous carbon are mainly introduced by direct pyrolysis, hydrothermal synthesis, and chemical etching. Peng et al. introduced carbon defects via pyrolysis of graphene oxide (GO) and boron nitride at 300–900 °C for 3 h. As the temperature increased from 300 to 800 °C, the I_D_/I_G_ ratio in Raman spectroscopy rose from 0.98 to 1.19. With further heating to 900 °C, this value increased only slightly to 1.20. Calculations of the free energy of O_2_ activation revealed that the electron‐mediated oxygen reduction activity on the catalyst surface first increased and then decreased with rising defect concentration. The defect concentration was optimal at a pyrolysis temperature of 800 °C, and the activation energy barrier for molecular oxygen was minimal. The sulfamethoxazole degradation rate increased by more than one times, and the mineralization efficiency improved by 79% after the introduction of carbon vacancies.^[^ ^139^ ^]^ In addition, Xia et al. used NaCl as a hard template to encapsulate the precursor, coordinated Zn^2+^ with 2,6‐diaminopyridine, and thermally decomposed at 1000 °C for 2 h in an Ar atmosphere to form carbon vacancies. Density functional theory (DFT) showed the enhancement of O_2_ adsorption and the reduction of the O‐O dissociation energy barrier.^[^ ^140^ ^]^ The direct pyrolysis process is simple and efficient, but the vacancy defects are uncontrollable. Yang et al. used 3‐Amino‐1,2,4‐triazole as a precursor and heated it at 600 °C for 3 h to obtain the initial material CN600. The carbon defects were introduced by hydrothermal treatment of CN600 at 180 °C for 12 h. The results showed that the introduction of carbon defects enhanced O_2_ adsorption, narrowed the bandgap, and promoted carrier separation, which increased the removal rate of tetracycline by 14.4%.^[^ ^141^ ^]^ Moreover, Wang et al. hydrothermally treated Sterculia scaphigera at 180 °C for 12 h, and then kept it at 900 °C in an Ar atmosphere for 2 h to construct carbon vacancies. The results indicated that H_2_O_2_ yield increased by about 19%.^[^ ^142^ ^]^ The hydrothermal approach is mild and controllable, but the vacancy density is limited, and the yield is low. Cattle bone powder was utilized as a raw material to be thermally decomposed at 950 °C under an N_2_ atmosphere, and then etched with HCl to induce carbon vacancies. The data demonstrated that the oxidation efficiency of As(III) reached nearly 100% after acid etching, while the unetched samples could hardly oxidize As(III), so it proved that defect exposure is a prerequisite for activity.^[^ ^143^ ^]^ Wang et al. selectively broke C‐N bonds to form carbon vacancies through water vapor etching on g‐C_3_N_4_ frameworks at 460 °C, and increased the H_2_O_2_ yield to 14.9 mmol·g^−1^·h^−1^.^[^ ^144^ ^]^ Furthermore, Chen et al. etched melamine with H_2_SO_4_ and then calcined it at 550 °C under N_2_ to form carbon vacancies. In situ DRIFTS analysis revealed a significant increase in the peak intensity of highly active O_2_ species on the surface, and confirmed that carbon vacancies improved O_2_ activation.^[^ ^145^ ^]^ Peng et al. developed a heterojunction catalyst by the introduction of carbon defects onto GO. The degradation rate of sulfamethoxazole reached 0.0252 min^−1^, and the removal rate approached 100% within 60 min.^[^ ^139^ ^]^ Chemical etching has high defect concentration and wide applicability, but the process is complex, and excessive etching may damage the carbon skeleton.

### 3.2 Heteroatom Doping

In the design of electron‐rich materials, heteroatom doping is a core strategy for modulation of O_2_ activation performance. The purpose of heteroatom doping is to regulate the electronic structure of materials, such as work function, Fermi level position, charge distribution, and local electron density, to enhance O_2_ adsorption, electron transfer, and O‐O bond cleavage efficiency. Fe‐based materials primarily optimize the redox activity of transition metal centers in specific coordination environments via heteroatom doping. Bi‐based materials enhance O_2_ adsorption and charge transfer through the lone pair electrons of Bi^3+^ and the strong localized polarization effects induced by doping. Cu‐based materials focus on the stability of low‐valent Cu^+^ and interfacial electron transfer. Porous carbon relies on the properties of doped atoms and the developed local charge environment within the carbon framework as active sites for O_2_ adsorption and activation.

The method of doping with heteroatoms differs from one material to another. Fe‐based materials are mainly doped with heteroatoms by chemical substitution, ball milling, and high‐temperature pyrolysis. Liu et al. reduced Fe^2+^ in FeSO_4_ to Fe via Mg powder, and formed a Fe‐Mg bimetallic structure. The results showed that the accumulation of H_2_O_2_ increased by 34.5 mgL^−1^ within 1 h after Mg doping compared to the single Fe system. The removal rate of 4‐chlorophenol increased by 91% within 60 min.^[^ ^146^ ^]^ The chemical substitution method is convenient and controllable, but the material stability is limited. Chen et al. mixed NaCl with micron ZVI (mZVI) at a molar ratio of 2% and ball‐milled the mixture at 550 rpm for 4 h in a ball mill, and successfully doped Cl into mZVI. Consequently, it increased the removal rate of sulfamethazine from 0.08 to 0.95 min^−1^.^[^ ^147^ ^]^ The ball milling process can efficiently introduce OVs with high stability, but the structural changes in the material are uncontrollable. Besides, Chen et al. used L‐cysteine with melamine and FeCl_3_ to thermally decompose at 550 and 800 °C for 2 h, and introduced S atoms into the second coordination layer of Fe‐N_4_. The data demonstrated that S doping increased the proportion of low‐spin Fe^3+^ by 29.2%, optimized the adsorption strength of the Fe center on OH^*^, reduced the O‐O bond energy from 2.0 to 1.9 eV, and promoted O─O bond cleavage.^[^ ^148^ ^]^ High‐temperature pyrolysis can form highly ordered crystal structures, but consumes high energy and may cause metal particle aggregation.

Bi‐based materials are mostly doped with atoms via hydrothermal/solvothermal processes and chemical precipitation. Hu et al. hydrothermally treated Bi citrate and NaBr at 180 °C for 12 h, and achieved doping through Br‐substitution of lattice oxygen sites. Br doping reduced the conduction band potential from −0.1 to −0.4 V versus NHE, and increased the removal rate of ciprofloxacin by 60% within 30 min.^[^ ^126^ ^]^ Moreover, Liu et al. used H_3_BO_3_ as the boron source and made it react with Bi(NO_3_)_3_·5H_2_O and cetyltriethylammonium bromide in EG at 160 °C for 16 h. Consequently, B^3+^ was introduced through lattice substitution to induce surface hydroxyl enrichment, and the removal rate of RhB increased by 28.2% within 30 min.^[^ ^124^ ^]^ The hydrothermal/ solventothermal method provides high crystallinity and precise control of doping levels, but it requires a long reaction time. Furthermore, Jia et al. dissolved Bi(NO_3_)_3_·5H_2_O in acetic acid solution and then added NaCl or NaBr and KI to form BiOCl and BiOBr precipitates, respectively. Subsequently, they further constructed I‐BiOCl/I‐BiOBr heterojunctions by deposition. The results showed that the materials transformed from I‐type heterojunctions to II‐type heterojunctions after I doping, which promoted the efficient migration of photo‐generated electrons to the conduction band of I‐BiOBr to facilitate O_2_ activation. The removal rate of phenol increased by ≈24% within 4 h.^[^ ^125^ ^]^ Additionally, BiOCl_x_Br_1‐x_ precipitate was formed by the dissolution of Bi(NO_3_)_3_ in acetic acid solution and the addition of cetyltrimethylammonium bromide (CTAB) and cetyltrimethylammonium chloride. The built‐in electric field between the [Bi_2_O_2_]^2+^ layer and the halogen layer promoted electron‐hole separation, and achieved complete degradation of RhB in 2 min.^[^ ^149^ ^]^ The precipitation process is easy to operate, and the morphology is controllable, but the distribution is uneven.

Heteroatoms are induced into Cu‐based materials through ion exchange and solvothermal methods. Pre‐synthesized Cu_2_O nanocubes were stirred in a RhCl_3_ aqueous solution for 6 h to introduce Rh. The current density reached 49.4 mAcm^−2^ at 1.6 V after Rh doping, and improved by about 100 times.^[^ ^130^ ^]^ The ion exchange method is uniform and precise in structure, but it has a low loading capacity. In comparison, the solvothermal method has a high active area and strong stability, but may introduce impurities. Xiong et al. put pre‐synthesized CuO nanoarrays in an ethylene glycol solution with Co(NO_3_)_2_ and reacted it at 120 °C for 2 h. The specific surface area of the Co‐doped material increased from 106.7 to 210.3 m^2^g^−1^, and it boosted the redox properties of CuO and lowered the activation energy barrier of O_2_.^[^ ^129^ ^]^

Porous carbon materials are mainly doped with heteroatoms through the template method, biomass pyrolysis, and stepwise carbonization. S─N co‐doped hierarchical porous carbon composite was prepared at 900 °C in an argon atmosphere for 2 h using melamine and thiocyanuric acid as precursors, CTAB as a surfactant template, and polyethyleneimine as a modifier. The results indicated that the charge transfer efficiency was improved after doping, and it was beneficial for O_2_ adsorption and activation.^[^ ^131^ ^]^ Besides, Jing et al. utilized GO as a template and copolymerized resorcinol‐ethylenediamine‐formaldehyde resin in a CTAB/1,3,5‐trimethylbenzene emulsion. After activation with ZnCl_2_, the material was carbonized at 800 °C to obtain graded porous carbon nanosheets co‐doped with N and O. The results revealed that the combination of C═O and graphite N formed the optimal active site C═O─Gra─N, with an adsorption energy of 4.3 eV for the intermediate ^*^OOH and an overpotential of only 0.06 V.^[^ ^132^ ^]^ The template method provides controllable pore structures and uniform distribution of heteroatoms, but the process is complex. Additionally, bovine serum albumin was hydrolyzed at 100 °C in a KOH solution for 12 h to produce protein fragments and carbon dots. After freeze‐drying, the material was pyrolyzed at 900 °C for 2 h to obtain co‐doped porous carbon with N and S. The specific surface area increased by nearly 10 times, and the overpotential decreased by ≈1.5 V, which is conducive to promoting O_2_ activation.^[^ ^150^ ^]^ Biomass pyrolysis is environment‐friendly but has poor conductivity and uncontrollable doping. Chen et al. impregnated biomass precursors with H_3_PO_4_, pre‐carbonized them at 600 °C, and then carbonized them again at 800 °C to get phosphorus‐doped porous carbon. The phosphorus doping lowered the overpotential (0.20 V) and increased the number of active sites by 35%.^[^ ^151^ ^]^ The stepwise carbonization process is simple and achieves high doping levels, but the high‐temperature pore structure is prone to collapse.

### 3.3 Edge Defect

Edge defects are localized defective regions at the surface or interface of a material due to termination of atomic arrangement, coordination unsaturation, or structural discontinuity. Such defects are usually characterized by a decrease in the number of atomic coordinations at the edge sites, an uneven charge distribution, or a reconfiguration of the electron density.^[^ ^139^, ^152^ ^]^ Electron‐rich materials overcome the O_2_ activation bottleneck through low‐coordination dangling bonds at edge defects. Fe‐based materials utilize the asymmetric structure of sawtooth edges or grain boundary steps to break the symmetry of Fe sites. This induces d‐orbital localization and spin reduction to weaken the O‐O bond energy barrier. Bi‐based materials rely on the dangling bonds of low‐coordination Bi atoms at the edges of nanosheets to drive space charge separation, and enrich electrons to inject into O_2_ to generate $O_{2}^{\cdot -}$. With twin boundaries, Cu‐based materials reduce the coordination number of Cu, induce lattice strain, and shift the d band center upward to stabilize the ^*^OOH intermediate. For porous carbon, sp^2^ dangling bonds at zigzag or armchair‐type edges inject π electrons, which enhance electron transfer efficiency in synergy with ultra‐microporous confinement.

Edge defects are constructed by various methods in different electron‐rich materials. Fe‐based materials incorporate edge defects via high high‐temperature and pressure method, or ligand self‐assembly method. A mixture of Fe and B powders was reacted at 5 GPa and 1300 °C for 20 min to induce stacking faults. The results showed that the d‐band center of Fe shifted from ‐2.0 to −1.8 eV after edge defects were introduced, and the rate‐determining step energy barrier decreased by 18.2%.^[^ ^153^ ^]^ The high‐temperature and pressure process offers a short reaction time and high material conductivity, but it requires sophisticated equipment, and the dislocation density is affected by fluctuations in pressure and temperature. Additionally, Lee et al. used tridentate phosphinimide ligands to react with Fe[N(SiMe_3_)_2_]_2_ in 2‐methylfuran to form low‐coordination trinuclear Fe clusters, which were activated by solid‐state O_2_ to create bridged structures. The bridged and terminal coordination modes of the phosphinimide ligands forced the Fe center to develop geometric gaps. The results indicated that low coordination enhanced the reduction ability, and drove multi‐electron transfer. High‐valent Fe oxide species could be directly generated for spontaneous O─O bond cleavage and small molecule activation.^[^ ^154^ ^]^ The ligand self‐assembly method can precisely induce defect geometric structures through ligands under mild conditions, but solid‐state reactions depend on crystal quality.

Edge defects in Bi‐based materials are primarily induced via a one‐step precipitation and electrochemical reconstruction process. Guo et al. dissolved Bi(NO_3_)_3_·5H_2_O in a mixture of mannitol and polyvinyl pyrrolidone, then slowly added NaBr to synthesize ultra‐small BiOBr quantum dots to expose abundant edge coordination unsaturated sites. The results showed that the $O_{2}^{\cdot -}$ yield increased from 47 µmol·L^−1^·h^−1^ to 166 µmol·L^−1^·h^−1^, and the charge transfer efficiency improved by 4.8%.^[^ ^155^ ^]^ Sun et al. achieved an ≈80% increase in the removal rate of bisphenol A via photocatalysis within 60 min after the introduction of edge defects in BiOCl.^[^ ^156^ ^]^ The one‐step precipitation is easy to operate with a high density of edge sites, but it may cause secondary pollution. Xu et al. synthesized BiOBr nanoplate precursors via hydrothermal synthesis, then converted BiOBr into Bi nanoplates via electrochemical reduction, and introduced edge defects. The results revealed an 80% reduction in charge transfer resistance and a 194.0 µF·cm^−2^ increase in electrochemically active area.^[^ ^157^ ^]^ Moreover, Lv et al. first obtained Bi_19_Br_3_S_27_ nanowire precursors via solvent‐thermal synthesis, and then removed Br while retaining S by electrochemical reconstruction to fabricate S‐modified Bi nanosheets with edge defects. The current density increased by ≈250 mA·cm^−2^, and the p‐band center near the S‐adjacent Bi sites shifted upward, which was beneficial for O_2_ activation.^[^ ^158^ ^]^ Electrochemical reconstruction can precisely control active sites, but the process is complex and material stability needs to be improved.

Cu‐based materials primarily build edge defects through heat treatment and electrochemical reduction. Zhang et al. used g‐C_3_N_4_ as a carrier, impregnated it with Cu(NO_3_)_2_ solution, and subjected it to thermal treatment at 250 °C to form Cu single‐atom catalysts anchored to the jagged edges of g‐C_3_N_4_. The results showed that edge defects optimized the coordination environment of Cu single atoms, enhanced Cu─O covalent bonding and d‐band center activity, and facilitated O_2_ activation.^[^ ^159^ ^]^ The thermal treatment is efficient and the material structure is stable, but the site selectivity is low and the structural control precision is insufficient. Li et al. synthesized polycrystalline Cu_2_O nanoparticles with grain boundaries by laser ablation of a Cu target in water. Pure Cu nanoparticles containing twisted nanotwins and edge dislocations were then formed by electrochemical reduction of Cu_2_O. The results indicated that the valence state of Cu atoms on the defect surface increased, and the electron transfer ability was enhanced. Furthermore, the upward shift of the d band center reinforced the electronic interaction between Cu and reaction intermediates.^[^ ^160^ ^]^ Electrochemical reduction can flexibly control the structure, but requires precise control of the electro‐reduction potential.

High temperature treatment is a necessary step in the construction of edge defects in porous carbon materials. According to different pretreatment methods, porous carbon materials can be constructed via acid oxidation, ionic liquid, fluorination‐defluorination cycle, or electrospinning process. Commercial multi‐walled carbon nanotubes were purified with HCl to remove metal impurities, then oxidized with HNO_3_ to introduce oxygen‐containing groups, and finally held at 920 °C for 30 min to generate jagged edge defects. The active surface area increased from 2.4 to 9.4 m^2^g^−1^, and the defect density improved by ≈36%.^[^ ^161^ ^]^ The acid oxidation can precisely construct active defects, but it is necessary to avoid excessive oxidation that could damage the structure. Wang et al. synthesized an ionic liquid by reaction of 1‐vinylimidazole with HNO_3_, which was treated at 900 °C for 1 h to remove N and form edge defects. The results revealed that the removal rate of naproxen was improved by 3.7 times, and the defect density was positively correlated with the removal rate.^[^ ^162^ ^]^ The ionic liquid method can precisely control the structure and defect type of porous carbon materials via the change of the anion type in the ionic liquid, but its stability needs to be improved. Lim et al. fluorinated activated carbon at room temperature to form covalent C─F bonds. Defluorination at 600–1200 °C reconstructed the active edge sites and introduced oxygen functional groups directly as O_2_ adsorption sites. Furthermore, the overpotential was reduced by 312 mV, and the catalytic efficiency per unit specific surface area was improved.^[^ ^133^ ^]^ The fluorination‐defluorination cycle can achieve selective activation of edge sites with abundant active sites, but it has a high equipment demand and may involve side reactions. Besides, Dong et al. prepared fiber precursors by electrostatic spinning with polyvinyl alcohol as the carbon source and polytetrafluoroethylene as the pore‐forming agent. Honeycomb‐shaped carbon nanofibers with abundant oxygen functional groups were obtained after carbonization. DFT calculations showed that edge defects at the junction of heptagons and pentagons could optimize the adsorption energy of ^*^OOH and lower the reaction energy barrier.^[^ ^163^ ^]^ Electrospinning offers controllable morphology and structure but involves a complex process with low yield.

## 4 Application of Electron‐Rich Materials‐Driven O_2_ Activation for Soil Remediation

According to the systematic analysis of the mechanisms and modification strategies for electron‐rich materials to activate O_2_, the potential application of these materials in practical soil remediation is significant. This section is devoted to exploring the application feasibility of metal minerals and carbon materials in soil remediation through O_2_ activation.

### 4.1 Metal Minerals‐Mediated Soil Remediation

**Figure 2:**
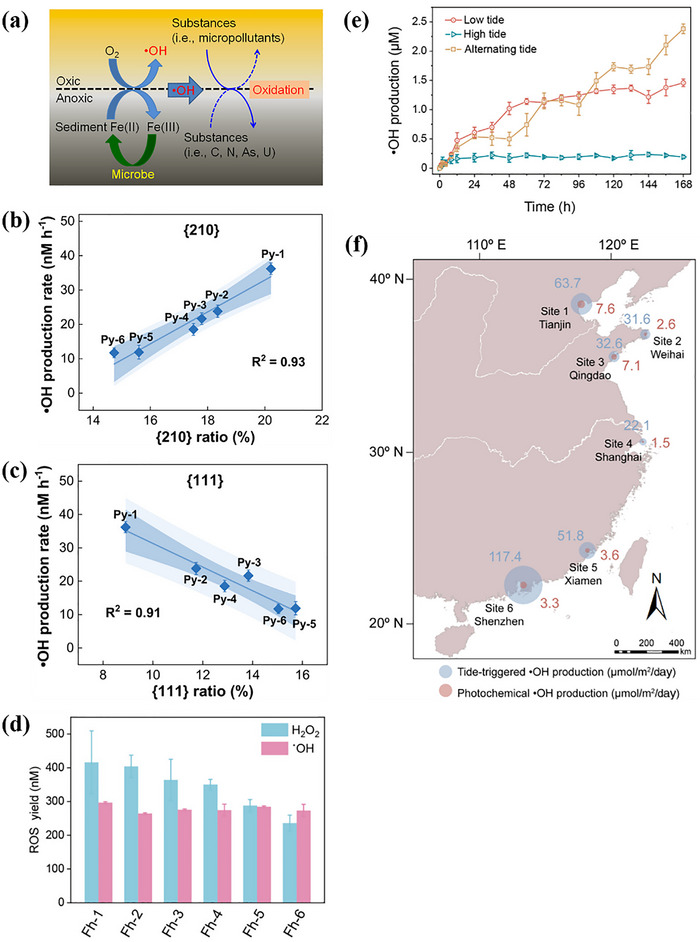
a) O_2_ activation mechanism of iron minerals under redox fluctuations in the underground environment. Adapted with permission. [167] Copyright 2015, American Chemical Society. Correlations between b) {210} and c) {111} facet ratios and ·OH productions. Adapted with permission.[34] Copyright 2023, American Chemical Society. d) Photochemical production of H_2_O_2_ (blue) by ferrihydrites with different crystallinities and ·OH productions (pink) from the reaction of ferrihydrites with H_2_O_2_ (1 mM) in aqueous solution under dark conditions. Adapted with permission. [35] Copyright 2024, American Chemical Society. e) ·OH production by pyrite incubated under high tide, low tide, and alternating tidal conditions. Adapted with permission. [169] Copyright 2023, American Chemical Society. f) Field site map showing area‐normalized ·OH production along the coast of China. Adapted with permission. [30] Copyright 2022, American Chemical Society.

As one of the most abundant redox‐sensitive minerals on the Earth's surface, Fe minerals in soil have the ability for O_2_ activation and play an important role in pollutant degradation and elemental cycle.^[^ ^40^, ^164^, ^165^, ^166^ ^]^ Previous researches on the O_2_ activation by Fe minerals mainly focused on the surface environment under illumination. In recent years, scholars have discovered the O_2_ activation from Fe minerals under redox fluctuations in the subsurface environment. Tong et al. found that Fe(II) minerals can convert O_2_ to H_2_O_2_ via electron transfer under aerobic conditions and generate ·OH via further Fenton‐like reactions. Meanwhile, microorganisms can reduce Fe(III) back to Fe(II) under anaerobic conditions to maintain the continuous ·OH production (**Figure** **2**a).^[^ ^167^ ^]^ Compared with adsorbed Fe(II) and ion‐exchangeable Fe(II), the mineral structure of Fe(II) has higher reactivity, and can activate O_2_ to produce ·OH via multi‐electron transfer. Zhang et al. found the ·OH yield of mineral‐structured Fe(II) could reach 2–3 times higher than that of adsorbed Fe(II), but ion exchange state Fe(II) could hardly contribute to ·OH generation through kinetic modeling. Due to the electron cloud density modulation, it is easier for electron acceptance and transfer, and thus significantly enhances the ROS generation efficiency from Fe(II) in the mineral lattice.^[^ ^168^ ^]^ Besides, the O_2_ activation from Fe minerals is influenced by the crystal surface, crystallinity, and tidal variation. The effect of pyrite crystal facet type on O_2_ activation was studied by Tan et al. As shown in Figure 2b, the higher the proportion of 210 crystal faces, the higher the ·OH yield, while the opposite was true for 111 crystal faces (Figure 2c). The ·OH yields from these two crystalline facets of pyrite differed by a factor of 3.1 in 48 h.^[^ ^34^ ^]^ Moreover, Wang et al. investigated the effect on the crystallinity of hydrotalcite for O_2_ activation. As shown in Figure 2d, the H_2_O_2_ concentration increased from 216 to 416 nM as the crystallinity decreased (from Fh‐6 to Fh‐1). Low‐crystallinity Fe minerals with many structural defects can enrich surface active sites to promote O_2_ activation.^[^ ^35^ ^]^ Therefore, efficient Fe‐based catalysts for soil remediation can be designed via modulation by the exposure crystal surface and crystallinity of Fe minerals. However, the comprehensive effect of crystal surface selection and crystallinity on catalytic stability and specific surface area needs to be evaluated. In addition, the redox oscillations triggered by tidal variations could offer a dynamic driving force for O_2_ activation from Fe minerals. It was demonstrated that the periodic redox fluctuations induced by tides could transform the high‐crystallinity pyrite into a lower‐crystallinity sub‐stable phase, and achieve a 2.4 times enhancement of ·OH production (Figure 2e).^[^ ^169^ ^]^ Hence, water supply through rotational irrigation can promote the regeneration of active Fe and strengthen the natural purification capacity of the soil. Furthermore, a field investigation of the coast within China was conducted by Zhao et al. It was revealed that coastal Fe minerals can generate ·OH (22.1–117.4 µmolm^−2^/day) by continuous O_2_ activation via tidal process. In comparison to the photocatalytic pathway, the ·OH output was enhanced by 5–36 times (Figure 2f).^[^ ^30^ ^]^ Therefore, tidal processes can reinforce the O_2_ activation from Fe minerals in coastal zone soils, improve the self‐purification capacity of nearshore soils, and reduce pollutant damage to ecosystems, which accelerates soil carbon and metal cycling. The intensification of natural processes such as tidal dynamics and seasonal wet‐dry alternation in coastal zone restoration can promote O_2_ activation by Fe minerals, and facilitate the reduction of artificial intervention costs. For instance, the process is expected to become a green procedure for wastewater treatment plants of coastal zones in the future, and will reduce the energy consumption of traditional UV or ozone processes.

**Figure 3:**
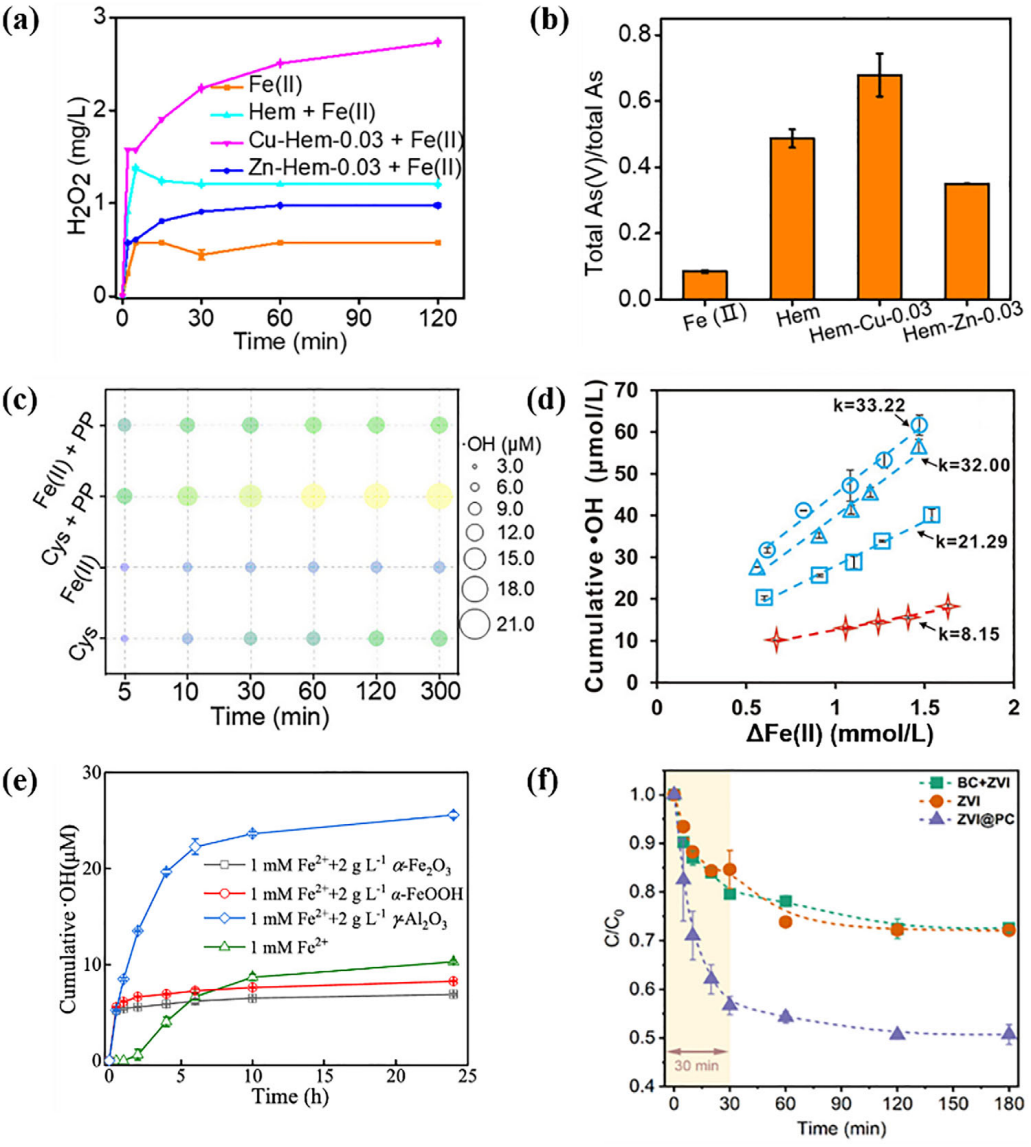
a) The H_2_O_2_ generation in Fe(II), Hem + Fe(II), Cu‐Hem‐0.03 + Fe(II), and Zn‐Hem‐0.03 + Fe(II) systems at pH 7.0. b) Total As(V)/total As after reaction in the presence of 5 mgL^−1^ Fe(II) at pH 7.0. Adapted with permission. [170] Copyright 2022, Elsevier. c) Cumulative accumulation of ·OH in Goe−Cys systems or Goe–Fe(II) systems under oxic conditions in the presence and absence of 2 mM PP. The solution pH was 7.0, the dosage of Fe oxyhydroxides was 0.5 gL^−1^, and the concentration of Cys was 2 mM. Adapted with permission. [71] Copyright 2024, American Chemical Society. d) Linear correlations between the ·OH yield and the amount of Fe(II) oxidized. Adapted with permission. [172] Copyright 2020, American Chemical Society. e) ·OH accumulation during oxygenation of Fe(II) in the presence of different minerals. Adapted with permission. [165] Copyright 2022, Elsevier. f) Kinetics of As(III) removal by ZVI@PC, ZVI, and BC+ZVI. Adapted with permission. [173] Copyright 2024, Elsevier.

For further improvement of the O_2_ activation efficiency of Fe minerals, scholars have investigated the synergistic activation of O_2_ by composite systems of Fe minerals and other substances. Fang et al. studied the remediation of As(III) contamination via Cu or Mg doping on the Fe mineral for O_2_ activation. It was shown that Cu‐doped hematite enhanced the cycle between Fe(II) and Fe(III) via the increase of oxygen vacancy density. Cu(II) could accept electrons from Fe(II) to form Cu(I), which then further transferred electrons to O_2_ and Fe(III) to form $O_{2}^{\cdot -}$ and Fe(II), respectively. Simultaneously, Cu(I) was converted back to Cu(II) to create a closed‐loop system. The H_2_O_2_ yield and As(III) conversion were improved by about 113.2% and 19.2% within 120 min, respectively, while those of Zn doping were reduced by around 30.8% and 13.7%, respectively (**Figure** **3**a,b).^[^ ^170^ ^]^ Moreover, Chen et al. realized efficient removal of hexachlorobenzene (HCB) from soil with Mg doping into ferrite minerals under microwave assistance. The results showed that the degradation and dechlorination rates of HCB were 80% and 93% within 20 min, respectively.^[^ ^171^ ^]^ The redox property, electronic structure and stability of Fe minerals can be tuned through the selection of specific metal doping. The choice of doping metal should be matched with the lattice and valence states of Fe mineral, and the screening of optimal doping elements can be guided by DFT calculation. Due to the complexity of the soil system, the synergistic influence of common organic and inorganic substances in the soil on the O_2_ activation by Fe minerals has been investigated. Chi et al. investigated the performance of cysteine (Cys) and pyrophosphate (PP) for the enhancement of pollutant degradation through synergy with pinnatite in a neutral environment. As shown in Figure 3c, compared with the Fe(II) system alone, the Cys and PP biligand system exhibited a 99.5%–125.7% improvement in ·OH generation, together with a ≈64% conversion of As(III).^[^ ^71^ ^]^ It was attributed to the fact that Cys, as an electron shuttle, can transfer electrons to Fe(III) for Fe(II) formation, and PP can reduce the thermodynamic potential barrier on the surface of Fe minerals. Furthermore, the mechanism of synergistic O_2_ activation between reduced Fe clay minerals (RIC) and HA was demonstrated by Zeng et al. A part of the structural Fe(II) in RIC can be solubilized by HA to form a water‐soluble Fe(II)‐HA complex for O_2_ activation via the homogeneous phase, and the ·OH yield of this pathway was nearly 4 times more efficient than that of the heterogeneous oxidation of RIC (Figure 3d). Simultaneously, the HA‐Fe(III) complexes could accept electrons from the structural Fe(II) of the RIC and realize the Fe(II)‐HA regeneration.^[^ ^172^ ^]^ The effect of Al_2_O_3_ on the O_2_ activation from Fe minerals under weak acid (pH = 6) was studied by Chen et al. As shown in Figure 3e, the ·OH production in the Fe(II)/Al_2_O_3_ system was elevated by 15.3 µmolL^−1^ in 24 h in comparison with the Fe mineral system alone.^[^ ^165^ ^]^ Therefore, for contaminated Fe‐rich soils, natural HA or exogenous low–cost ligands can be added to synergize with the Fe minerals for O_2_ activation to oxidize the pollutants in neutral conditions, while Al_2_O_3_‐based inorganic minerals or acid‐resistant ligands can be applied under weak acidic conditions. In situ remediation with low cost and low environmental disturbance is expected. ZVI was used for efficient activation of O_2_ to immobilize As(III) in soil by encapsulation of ZVI within a porous biomass carbon shell (ZVI@PC). The carbon shells could prevent soil particles and organic matter from contacting ZVI and promote the sustained release of Fe(II). Compared with single ZVI, the removal efficiency of As(III) by ZVI@PC at pH 4 for 30 min was improved by about 30% (Figure 3f). Meanwhile, the total arsenic in the effluent was reduced by 79.5% by 10 years of acid rain input simulated through the soil fixed bed.^[^ ^173^ ^]^ Therefore, the composite is suitable for heavy metal remediation of acidic soils around mining areas and is expected to achieve large‐scale industrial application in the future. Furthermore, the integration of Fe minerals with microbial fuel cells can enhance the in situ remediation of organic pollutants in soil. Chen et al. found that the degradation of total petroleum hydrocarbons in microbial fuel cell systems was significantly improved by Fe minerals. Among these, ferrihydrite demonstrated the most pronounced enhancement effect, and the removal rate of petroleum hydrocarbons increased by 74%.^[^ ^174^ ^]^

Notably, soil microorganisms play a crucial regulatory role in the redox cycle of metallic minerals. Soil microorganisms can utilize high‐valent metal minerals as electron acceptors under anaerobic or microaerophilic conditions and promote mineral reduction through extracellular electron transfer. These reduced intermediates can further participate in O_2_ activation to facilitate ROS generation. Han et al. demonstrated that the reduction from Fe(III) to Fe(II) by *Shivella* MR‐1 in anaerobic conditions could promote the conversion of oxidized solid organic matter into reduced forms. This process further boosted O_2_ activation to produce H_2_O_2_ and ·OH.^[^ ^175^ ^]^ Tong et al. showed that microorganisms could effectively reduce Fe(III) in sediments. The addition of microorganisms increased the concentration of Fe(II) in surface sediments from 0.5 to 0.9 gkg^−1^ via biological reduction.^[^ ^167^ ^]^ Moreover, current research has confirmed that soil microorganisms can enhance ROS production in metal minerals, and improve pollutant removal rate. Dai et al. indicated that heterotrophic bacteria released extracellular electrons through respiration, which were captured and stored by Fe minerals in the soil. These electrons were subsequently transferred to O_2_ to activate ROS production. In the presence of heterotrophic bacteria, ·OH production increased approximately four times.^[^ ^31^ ^]^ Wu et al. exhibited that the addition of strain JD37 could strengthen the Fe(II)/Fe(III) cycle and enhance the Fenton‐like reaction to generate ·OH. The removal rate of 2,4,4′‐trichlorobiphenyl increased by ≈45%.^[^ ^176^ ^]^

**Figure 4:**
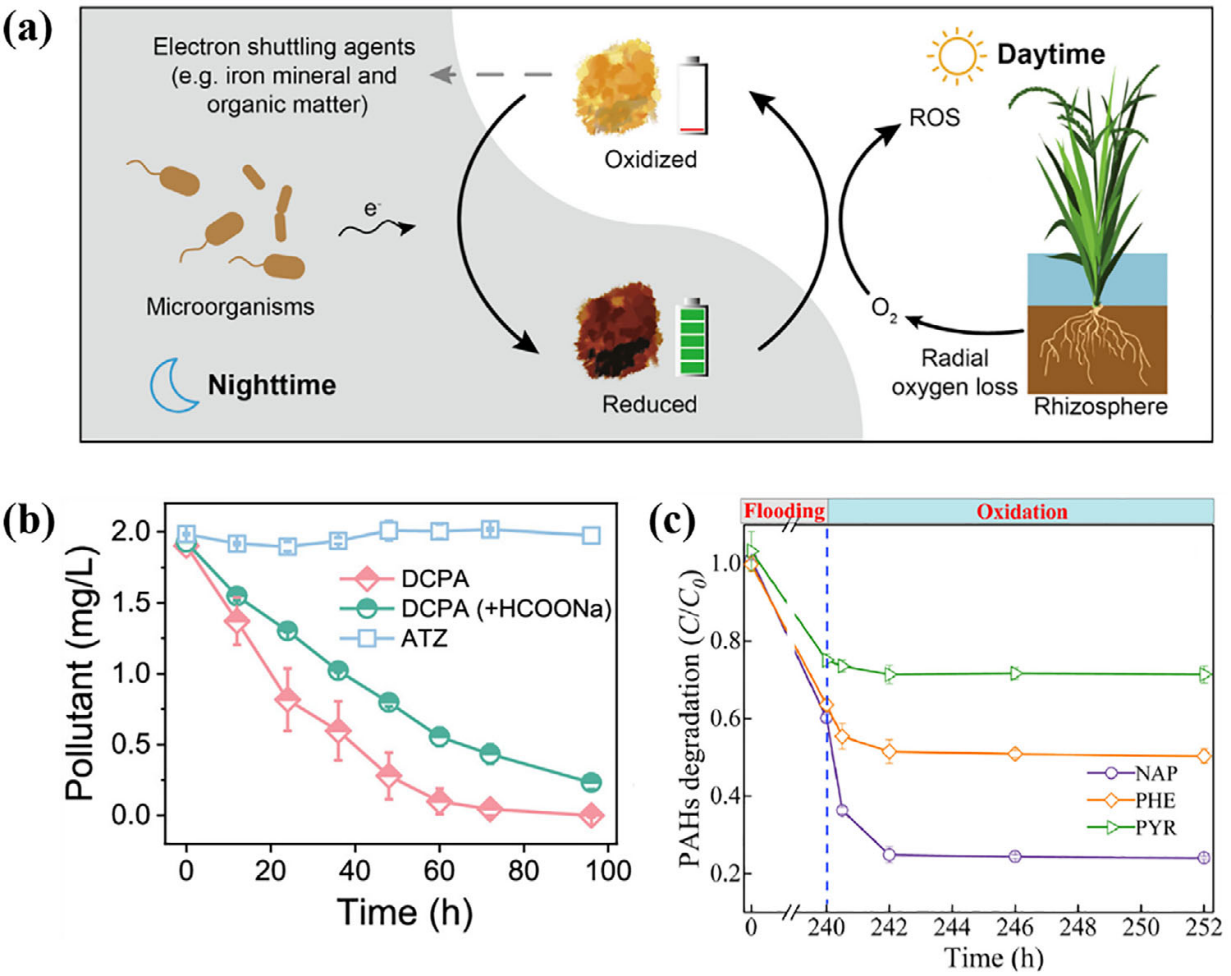
a) Schematic model for rhizosphere ROS production. Adapted with permission. [31] Copyright 2022, American Chemical Society.b) Removal efficiencies of diclofenac (DCPA) and atrazine (ATZ) in the rice rhizosphere and the molecular structures of DCPA and ATZ. Adapted with permission. [36] Copyright 2023, American Chemical Society. c) PAHs degradation during the redox fluctuations processes in JN_0‐20_ slurry. Adapted with permission. [33] Copyright 2021, Elsevier.

Recently, the mechanism of O_2_ activation by Fe minerals in plant roots has been evaluated. As shown in **Figure** **4**a, Dai et al. revealed that Fe minerals in the inter‐root acted as natural electron shuttles. It can receive extracellular electrons released from microbial respiration and transfer the electrons to O_2_ to drive the generation of $O_{2}^{\cdot -}$ and ·OH.^[^ ^31^ ^]^ Thus, inter‐root Fe minerals can facilitate the retention of natural contaminants. The risk of contamination of deep soil or groundwater can be reduced by regulating soil Fe mineral content. Currently, O_2_ activation via Fe minerals in plant roots has been applied for pollutant degradation in soil. Moreover, Meng et al. studied the degradation of interfacial herbicides with O_2_ activated by inter‐root Fe minerals in rice. The results showed that 2 mgL^−1^ of the more hydrophobic diclofenac was almost completely degraded within 100 h, while the less hydrophobic atrazine was hardly decomposed (Figure 4b).^[^ ^36^ ^]^ Therefore, due to the competitive adsorption of organic matter in organic matter‐rich soils, a combination of microbial metabolism or other auxiliary tools is needed to enhance the purification of hydrophobically weak pollutants. Besides, Chen et al. investigated the degradation of PAHs by Fe minerals in redox fluctuations in paddy soil. As shown in Figure 4c, the microbial degradation of naphthalene (NAP), phenanthrene (PHE) and pyrene (PYR) under 240 h anaerobic conditions was 39.9%, 36.4%, and 24.9%, respectively. After further exposure to O_2_ for 12 h, 36.1% of NAP was further degraded, which was much higher than PHE (13.2%) and PYR (3.7%). The results indicate that NAP and PHE are more susceptible to degradation by ·OH compared to PYR.^[^ ^33^ ^]^ It is possible that the stable conjugated system of PYR is more resistant to ·OH oxidation. In summary, inter‐root Fe minerals have broad‐spectrum degradation potential for a wide range of pollutants by activating O_2_ to produce ROS. However, their effects are significantly modulated by the nature of pollutants and redox fluctuations. Pollutants with strong hydrophobicity and simple structures are more likely to be efficiently degraded via radical‐dominated interfacial reactions, whereas pollutants with complex structures should rely on a multi‐stage redox synergistic or microbial‐assisted complex mechanism.

Currently, the research on direct activation of O_2_ through Bi‐based and Cu‐based materials for pollutant removal in soil remediation is lacking. The feasibility of soil application is merely explored in aqueous systems, but it holds potential for soil remediation. The limitations on mass transfer of O_2_ in the multiphase soil system, multi‐level interfacial electron transfer, and restricted light response require further exploration in the subsequent studies.

### 4.2 Carbon‐Mediated Soil Remediation

**Figure 5:**
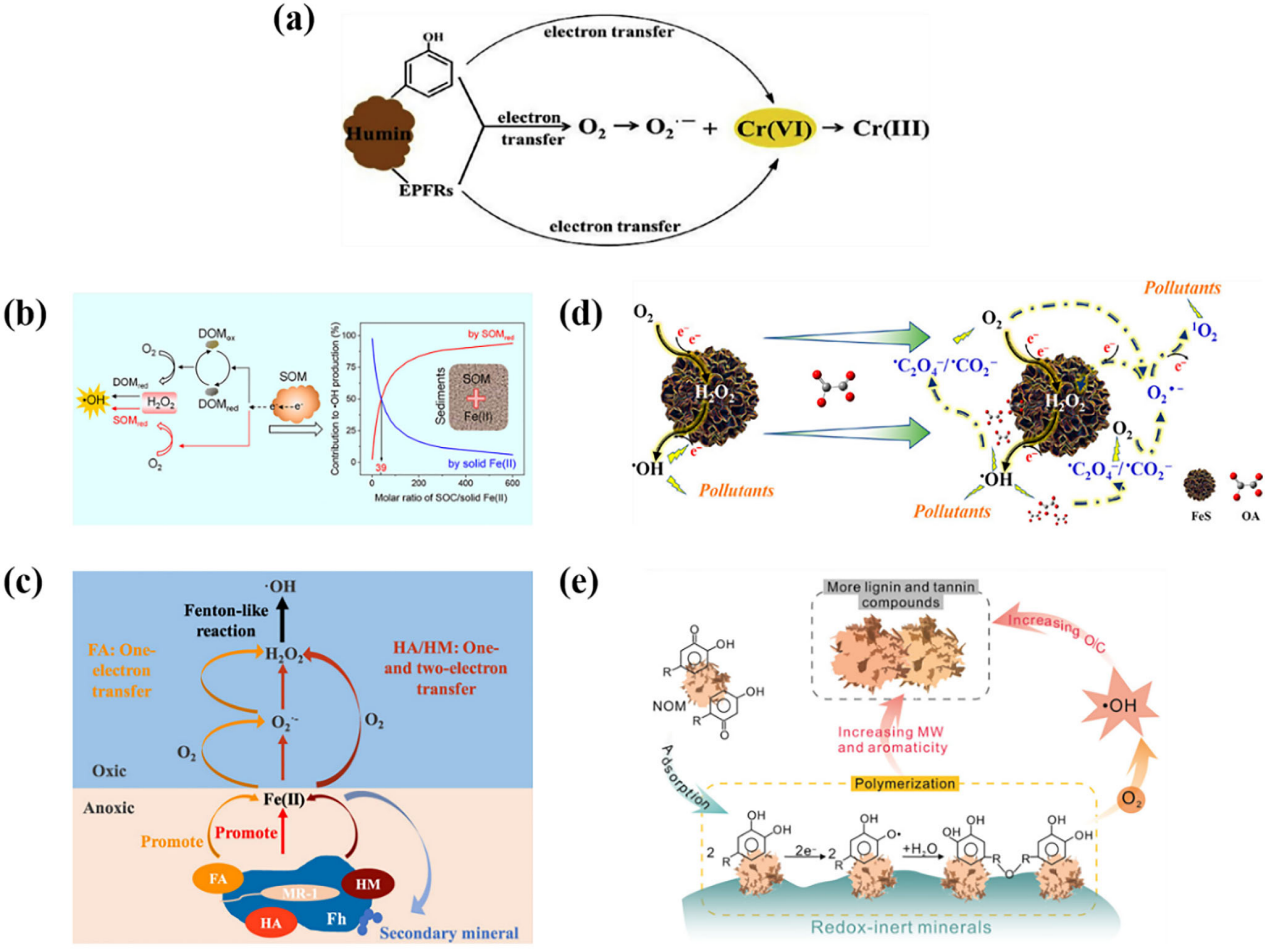
a) Reduction mechanism of hexavalent chromium by humus‐activated O_2_. Adapted with permission. [180] Copyright 2020, Elsevier. b) Mechanism of organic matter activation for O_2_. Adapted with permission. [32] Copyright 2022, American Chemical Society. c) Mechanism of O_2_ activation by hydrotalcite and organic acids. Adapted with permission.[175] Copyright 2022, American Chemical Society. d) Proposed pollutant transformation mechanism associated with OA. Adapted with permission. [181] Copyright 2023, American Chemical Society. e) Conceptual model of ⋅OH production and organic matter transformation induced by adsorption of oxidized HA onto redox‐inert minerals. Adapted with permission. [182] Copyright 2025, Elsevier.

Humus (HM) is an important component of soil with abundant reducing functional groups. It can activate O_2_ to form ROS such as $O_{2}^{\cdot -}$, ^1^O_2_, and ·OH, and plays an important role in pollutant degradation, soil carbon cycling, and microbial‐mediated redox processes.^[^ ^177^, ^178^, ^179^ ^]^ It has been reported that reduced‐state HM is one of the important contributors to O_2_ activation. Xu et al.^[^ ^180^ ^]^ investigated the mechanisms of O_2_ activation by a single HM based on Cr(VI) reduction under acidic conditions (**Figure** **5**a). The results showed that phenolic hydroxyl groups and environmentally persistent free radicals (PFRs) in HM can be used as electron donors to react with Cr(VI) directly. Moreover, it can also transfer electrons to O_2_ to produce $O_{2}^{\cdot -}$ for Cr(VI) reduction. Furthermore, the mechanisms of O_2_ activation by humic acid (HA) in a neutral environment have been explored (Figure 5b). It was found that O_2_ could be activated to H_2_O_2_ via double electron transfer from the hydroquinone group on the surface of solid HA. Meanwhile, the cycle of dissolved HA between the oxidized and reduced states could mediate the electron transfer between solid HA and O_2_ for ·OH generation.^[^ ^32^ ^]^ As a result, O_2_ is activated from HM in acidic and neutral environments primarily through one‐electron and two‐electron transfers, respectively. Since a large amount of Fe minerals are also present in the soil, subsequent studies also investigated the interaction between HM and Fe minerals. Han et al. revealed that the complexation between HA and FA and hydrotalcite inhibited the transformation from hydrotalcite to high crystallinity Fe oxides through the O_2_ activation with HA and fulvic acid (FA) at pH 7 (Figure 5c).^[^ ^175^ ^]^ Li et al. studied the degradation of bisphenol A (BPA) via O_2_ activation at pH 7 from oxalic acid (OA) and machinolite. As shown in Figure 5d, under the attack of ·OH initially oxidized by FeS, OA can produce carbon‐centric radicals (e.g., ^·^C_2_O_4_^−^ and ^·^CO_2_^−^) to activate O_2_ to produce the intermediates H_2_O_2_ and ^1^O_2_. Highly efficient removal of BPA (94%) was achieved via the subsequent Fenton‐like reaction for ·OH generation.^[^ ^181^ ^]^ Therefore, the presence of HM can inhibit Fe mineral crystallization and promote Fe(II)/Fe(III) cycling. Compared with HA and MA, the interaction between OA and Fe minerals can promote the generation of ^1^O_2_, and is more suitable for the remediation of soils contaminated by dyes and chlorophenols. The O_2_ activation by oxidation state HM on the interface of silica‐aluminate inert minerals in a neutral environment has recently been studied (Figure 5e). It was shown that the oxidation state of HM adsorbed by aluminum minerals could promote the generation of semiquinone radicals through electron transfer from polyphenols on HM. The semiquinone radicals then undergo a coupling reaction to form polymers, and thus enhance the electron‐donating capacity of HM for further O_2_ activation.^[^ ^182^ ^]^ In conclusion, HM exhibits strong redox state adjustability. In acidic or anoxic environments, it is more likely to exist in the reduced state. It can directly serve as an electron donor to reduce O_2_, and may be suitable for short‐term soil remediation. On the other hand, in neutral or weakly oxidized environments, HM exists in the oxidized state. It relies on mineral interface‐induced structural remodeling to form a more stable electron transfer network. This promotes soil carbon stabilization and may be applicable for long‐term soil remediation.

**Figure 6:**
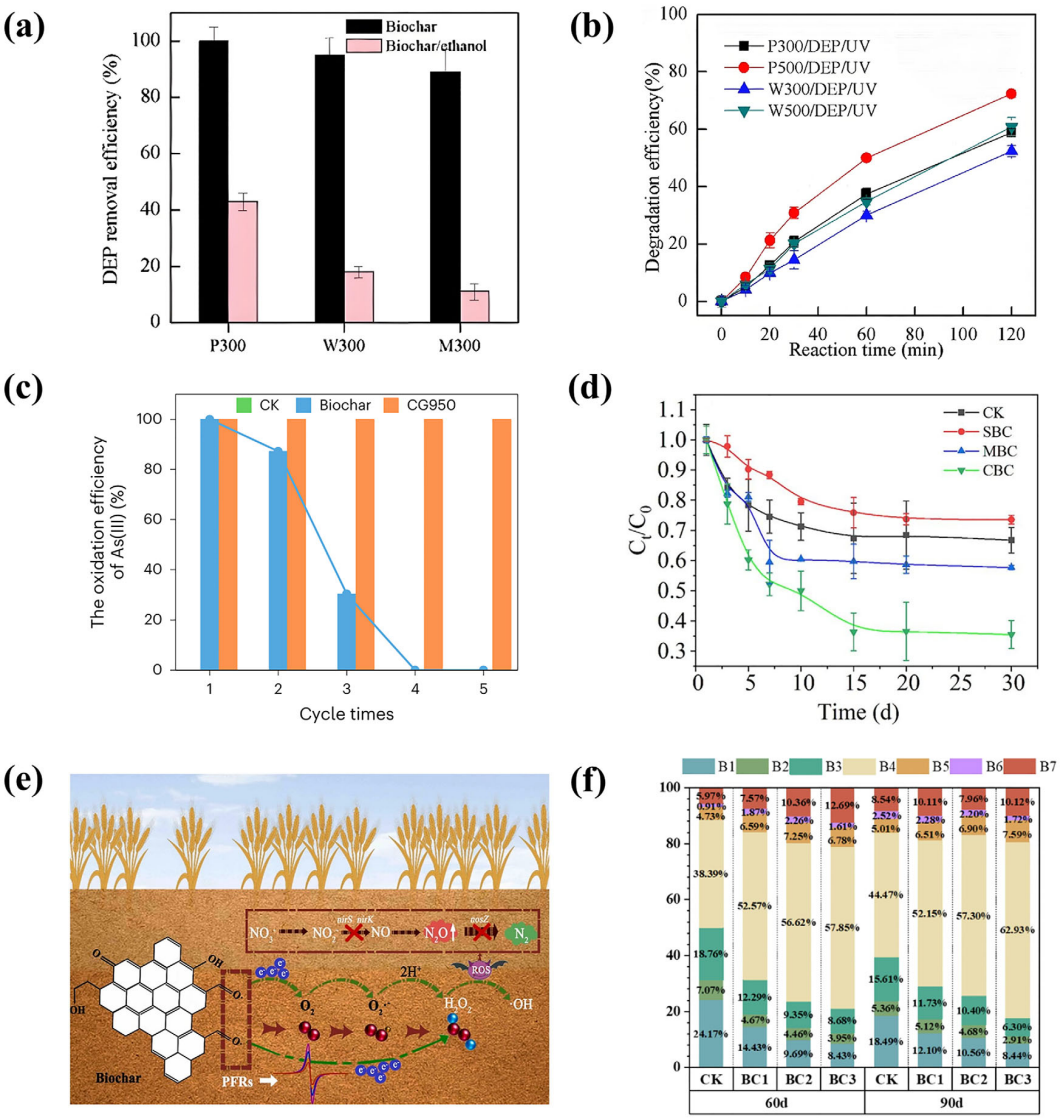
a) Degradation of DEP in biochar suspensions with purging of oxygen at 0.2 mLmin^−1^. Adapted with permission. [183] Copyright 2015, Elsevier. b) Photodegradation of DEP under UV light in the presence of different biochar particles. Adapted with permission. [184] Copyright 2017, Elsevier. c) The As(III) oxidation efficiency of CG950 and biochar during five charging and discharging cycles. Adapted with permission. [143] Copyright 2024, Springer Nature. d) The degradation of DBP in red soil treated with biochar. Adapted with permission. [185] Copyright 2025, Elsevier. e) Schematic diagram of ROS generation by O_2_ revitalized via biochar to inhibit N_2_O emission. Adapted with permission. [186] Copyright 2023, Elsevier. f) Effects of biochar addition amount (1 %, 2 %, and 3 % w/w) and remediation time (60 d and 90 d) on the proportions in soils. Adapted with permission. [187] Copyright 2025, Elsevier.

Biochar is primarily produced through biomass pyrolysis in an oxygen‐depleted environment. It can activate O_2_ to generate ROS, and directly attack pollutants to remediate soil. Previous studies have investigated the interfacial reactions involved in biochar‐activated O_2_ to generate ROS for soil pollution remediation. Fang et al. showed that PFRs from pine needle biochar could transfer electrons to O_2_ to generate ·OH radicals, and achieved a 100% removal of diethyl phthalate (DEP) within 24 h (**Figure** **6**a).^[^ ^183^ ^]^ Moreover, Fang et al. investigated the removal efficiency of DEP by biochar under UV irradiation. Results indicated that O_2_ could be activated via photochemical processes of dissolved organic matter, quinones in the biochar carbon matrix, and PFRs to generate ·OH and ^1^O_2_ mostly. As shown in Figure 6b, pine needle biochar achieved a 72.3% removal of DEP within 2 h.^[^ ^184^ ^]^ In addition, Fang et al. studied the removal efficiency of As(III) by O_2_ activation from bovine bone biochar. Complete oxidation of As(III) was achieved within 6 h at pH 7.5, with oxidation efficiency remaining at 100% after five cycles (Figure 6c). Notably, bovine bone biochar exhibited broad‐spectrum activity, and the removal rate of organic pollutants such as dyes, antibiotics, and brominated flame retardants reached 100% in 6 h.^[^ ^143^ ^]^ Therefore, the above studies demonstrate that biochar can rapidly and efficiently activate O_2_ to generate ROS for pollutant removal under controlled conditions, and highlight its high application value as a green carbon material in soil remediation. More importantly, these interfacial mechanisms need to be validated in real soil environments. Recently, scholars have explored the pollutant removal capabilities of biochar in soil environments. Li et al. showed that PFRs on the surface of cotton straw biochar can activate O_2_ to produce ·OH. After the addition of cotton stalk biochar, DBP removal efficiency reached 64.5% within 30 d and improved by 31.2% (Figure 6d).^[^ ^185^ ^]^ It proved the feasibility of biochar‐based soil remediation. However, compared with interfacial reactions, the remediation efficiency was lower and required a longer time. This reflects the applicability of field application, but the impact of soil complexity on ROS stability needs to be addressed. Noticeably, while the direct ROS‐generating effect of biochar benefits pollution remediation, it may also trigger adverse effects. Wu et al. evaluated the impact of biochar‐derived ROS on soil N_2_O emissions and their potential mechanisms. As shown in Figure 6e, PFRs in straw biochar could activate O_2_ to generate ROS via electron transfer. In particular, H_2_O_2_ and ·OH could inhibit microbial N_2_O reductase, and weaken biochar's ability to reduce N_2_O emissions.^[^ ^186^ ^]^ Therefore, the appropriate elevation of pyrolysis temperature facilitates the formation of highly graphitized structures and enhanced conductivity to promote $O_{2}^{\cdot -}$ generation via electron transfer. Furthermore, it is important to introduce specific heteroatoms to alter the adsorption patterns and reduction pathways of O_2_ on the carbon framework and boost ^1^O_2_ production. Simultaneously, the dispersion of active sites via the construction of a developed mesoporous structure can avoid excessive local ROS concentrations that may cause undue stress to microorganisms. This approach balances remediation efficiency with greenhouse gas reduction while avoiding potential ecological risks. The remediation capacity of biochar is not limited to direct ROS generation but also involves indirect mechanisms. Zhu et al. utilized lignin biochar as an electron shuttle to mediate the one‐electron transfer from ascorbic acid to O_2_, and the removal rate of BPA (20 ppm) reached 54% after 120 h.^[^ ^55^ ^]^ Moreover, Jia et al. demonstrated that corn stover biochar could activate O_2_ to generate $O_{2}^{\cdot -}$, and promote the transformation of Cr(VI) forms in soil. This increased plant uptake of water‐soluble Cr(VI), adsorbed Cr(VI), and precipitated chromate Cr(VI). As a result, the Cr removal rate improved by 14.5% and reached 80.6% after 90 d with 3% biochar addition (Figure 6f).^[^ ^187^ ^]^ Hence, biochar can be used for soil remediation by mediating O_2_ activation. Although its efficiency is slightly low, its diversified mechanisms and synergistic systems broaden its application scope, and provide flexibility for optimization of soil remediation strategies.

## 5 Effects of Different Factors on Soil Remediation by O_2_ Activation

Soil type is the fundamental factor determining its physical structure, chemical composition, and biological activity. Based on texture, soils can be classified into sandy soil, loam soil, and clay soil. Different soil types exhibit variations in O_2_ mass transfer, the contact state at soil interfaces, and interfacial electron transfer (**Table** **2**).^[^ ^188^, ^189^, ^190^ ^]^

**Table 2:** Differences in O_2_ mass transport and interfacial electron transfer across various soil types.

Soil type	Physicochemical properties	O_2_ transport	Interfacial environment	Electron‐transfer characteristics	Refs.
Sandy soil	Large pores, low CEC, low OM content	Fast	Loose interface, weak adsorption, low water content	Discontinuous pathways and weak electron supply	[188, 191, 192]
Loam soil	Balanced texture, moderate OM content, stable structure	Moderate	Moderate adsorption, coordinated mass transfer and reaction	Moderate electron transfer, electron‐shuttling may occur	[193, 194, 195]
Clay soil	Small pores, high water content, high CEC, rich in reactive minerals	Slow	Highly heterogeneous interface, strong adsorption	Strong electron‐buffering capacity, interfacial electron transfer active	[189, 190, 196]

OM: Organic matter; CEC: Cation exchange capacity.

### 5.1 Soil pH

**Figure 7:**
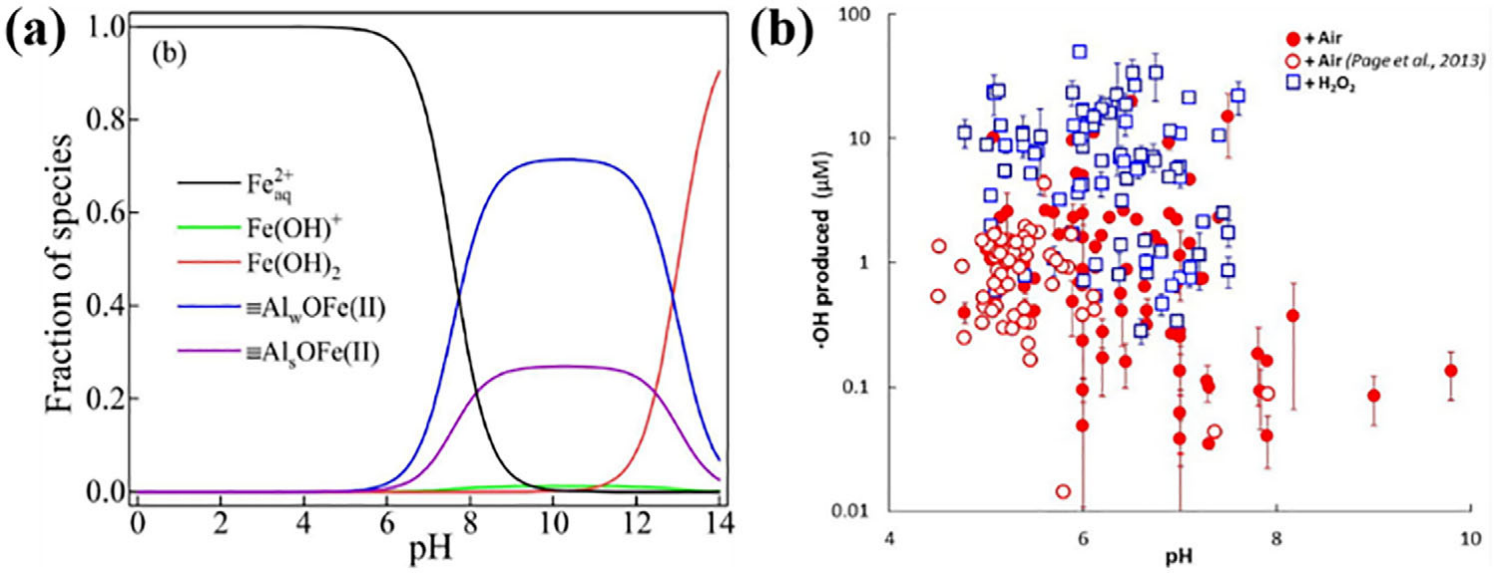
a) Fe(II) species in aqueous solution as a function of pH. Adapted with permission. [165] Copyright 2022, Elsevier. b) ⋅OH production from oxidation by O_2_ as a function of initial pH of the water, including surface and soil water, and soil leachates. Adapted with permission. [197] Copyright 2018, Elsevier.

As a key regulator, soil pH can affect the surface chemistry, Fe(II)/Fe(III) cycle efficiency and ROS generation pathway of metal minerals. Thus, it affects pollutant degradation efficiency and heavy metal remediation outcomes (**Table** **3**). Under acidic conditions (pH 5–6), Fe(II) exists mainly in the dissolved state and becomes more susceptible to successive reductions of O_2_ through electron transfer in the homogeneous Fenton reaction. Chen et al. showed that Fe(II) adsorption increased in the Fe(II)/α‐Al_2_O_3_ acidic system, and the efficiency of the reaction between surface Fe(II) and O_2_ for H_2_O_2_ generation was improved, resulting in a significant enhancement of ·OH generation by the Fenton reaction. With pH raised from 6 to 8, Fe(III) generated from the oxidation of Fe(II) was hydrolyzed to generate the low‐activity hydroxyl oxide α‐FeOOH (**Figure** **7**a), and the subsequent generation of ROS was inhibited.^[^ ^165^ ^]^ Moreover, Trusiak et al. demonstrated that sustained oxidation of Fe(II) in Arctic acidic soil water (pH≈6) caused ·OH accumulation to be significantly higher than in neutral or alkaline environments (pH>7.5) (Figure 7b).^[^ ^197^ ^]^ Therefore, low pH can promote Fe(II) oxidation and ROS generation.

**Table 3:** Pollutant degradation through O_2_ activation by electron‐rich material.

Material	Dosage	Reaction time	pH	Main ROS	Pollutant	Initial concentration	Efficiency (µmol·g^−1^·h^−1^)	Ref.
Fe minerals	100 gL^−1^	6 h	7	·OH	TCE	7.6 µmolL^−1^	0.004	[168]
Pyrite	0.5 gL^−1^	24 h	7	H_2_O_2_, $O_{2}^{\cdot -}$, ·OH	CBZ	1 µmolL^−1^	0.07	[34]
Pyrite	0.5 gL^−1^	24 h	7	H_2_O_2_, $O_{2}^{\cdot -}$, ·OH	BPA	1 µmolL^−1^	0.06	[34]
Pyrite	0.5 gL^−1^	24 h	7	H_2_O_2_, $O_{2}^{\cdot -}$, ·OH	Phenol	1 µmolL^−1^	0.08	[34]
Hydrotalcite	0.1 gL^−1^	2 h	7	H_2_O_2_, ·OH	Phenol	1 µmolL^−1^	4.8	[35]
Hydrotalcite	0.1 gL^−1^	2 h	7	H_2_O_2_, ·OH	Metoxuron	1 µmolL^−1^	4.6	[35]
Hydrotalcite	0.1 gL^−1^	2 h	7	H_2_O_2_, ·OH	Isoproturon	1 µmolL^−1^	4.4	[35]
Hematite	1 gL^−1^	2 h	7	H_2_O_2_	As(III)	5 mgL^−1^	16.3	[170]
α‐Fe_2_O_3_ +γ‐Al_2_O_3_	2.0 gL^−1^	4 h	6	·OH	Phenol	4.8 µmolL^−1^	0.3	[165]
ZVI@PC	0.5 gL^−1^	3 h	4	·OH, $O_{2}^{\cdot -}$	As(III)	20 mgL^−1^	76.4	[173]
Hydrotalcite	/	96 h	5.5	·OH,	Propanil	2 mgL^−1^	/	[36]
Hydrotalcite	3.6 gkg^−1^	12 h	6.6	·OH	NAP	5 mgL^−1^	/	[33]
Humic acid	25 gL^−1^	168 h	2	$O_{2}^{\cdot -}$	Cr(VI)	2 mmolL^−1^	0.3	[180]
Oxalic acid	1.5 mM	4 h	7	H_2_O_2_, ·OH	BPA	44 µmolL^−1^	76.6	[181]

BPA: Bisphenol A; CBZ: Carbamazepine; TCE: Trichloroethylene; NAP: Naphthalene. “/” represents Not Available.

In the neutral to weakly basic range (pH 7–8), the solubility of Fe(II) decreases, and the oxidized Fe(III) is prone to form colloids or precipitates. ROS generation is dominated by a heterogeneous reaction pathway, and Fe(II) adsorbed on the mineral surface can directly reduce O_2_ to $O_{2}^{\cdot -}$ by single electron transfer. This subsequently triggers a disproportionation reaction to produce H_2_O_2_, generating ·OH at the mineral interface. The crystallinity of Fe minerals and the percentage of adsorbed state Fe(II) become critical for ROS generation at pH 7–8. Zhao et al. found that the weakly crystalline Fe(II) phase of acicular ferrite was produced by microbial reduction in an alternating tidal environment at pH 7. And it can produce H_2_O_2_ efficiently through direct O_2_ activation from surface adsorbed state Fe(II) in the oxidized phase, while the low proportion of dissolved state Fe(II) leads to the limitation of the conventional Fenton pathway.^[^ ^169^ ^]^ Therefore, this mechanism can explain the much higher ·OH yield at low tide (≈1.4 µmolL^−1^) than the dissolved state Fe(II)‐dependent ·OH yield at high tide (≈0.2 µmolL^−1^).

At pH>8, the oxidation rate of Fe(II) increases abruptly and is converted to highly crystalline Fe(III) oxides. Its weak electron transfer capacity and reduced surface adsorption sites lead to a drastic decrease in ROS generation. Chi et al. altered the surface groups of acicular ferrite by complexation of Fe minerals with Cys and PP ligands, reducing the negative effect of the negative surface charge directly. Furthermore, complexation of the ligands could accelerate the dynamic electron transfer pathway and lower the energy barrier for O_2_ adsorption.^[^ ^71^ ^]^ The negative charge on the surface of Fe minerals is enhanced under alkaline conditions due to deprotonation of hydroxyl groups. Therefore, synergistic O_2_ activation by Cys and PP in Fe minerals under alkaline conditions can be attempted in the future, and it is expected to partially counteract the adverse effect of high pH through functionalized ligands on the mineral surface.

### 5.2 Dissolved Oxygen Content in Soil

**Figure 8:**
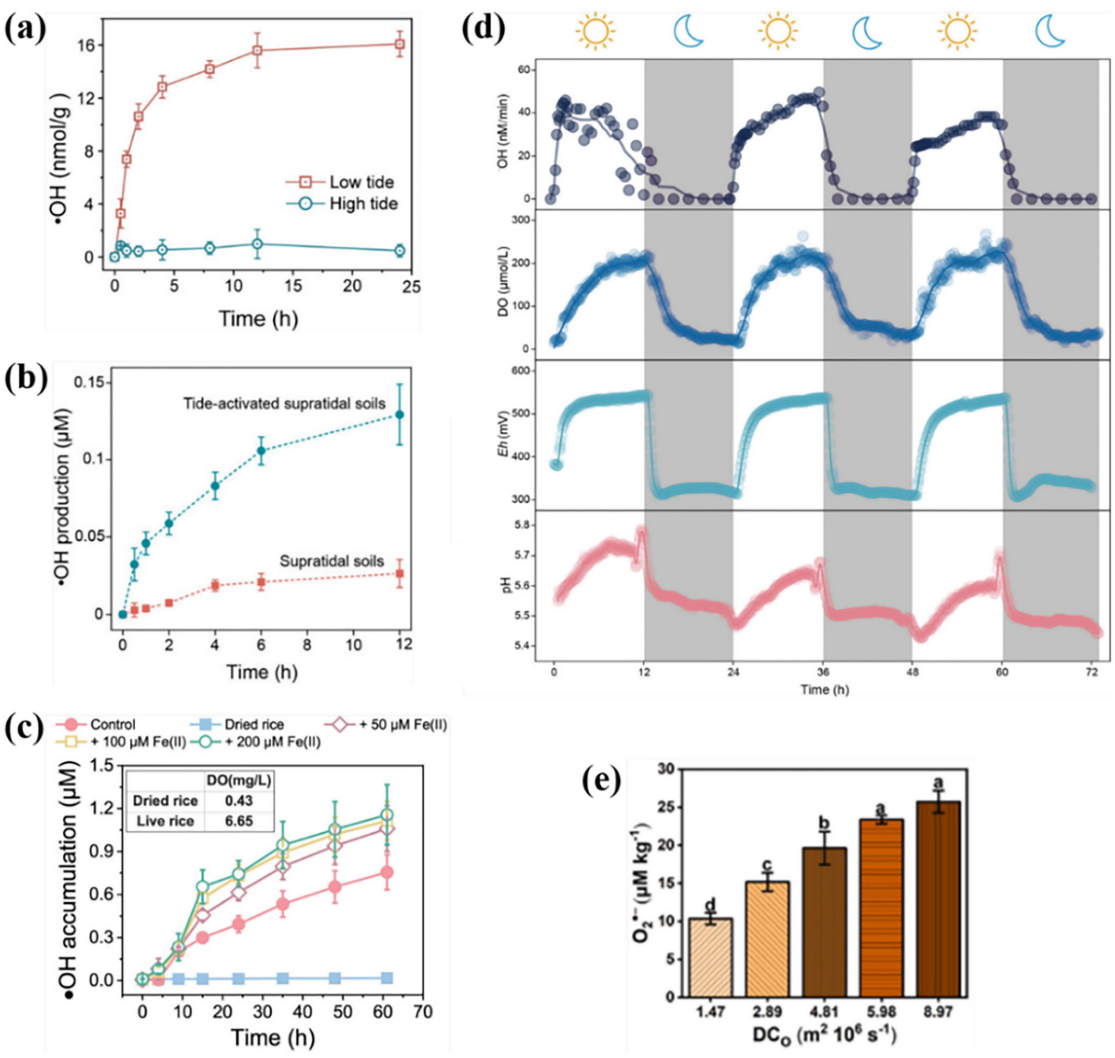
a) Time series of ⋅OH production. Adapted with permission. [30] Copyright 2018, Elsevier. b) Enhanced ⋅OH production in tide‐activated supratidal soils. Adapted with permission. [169] Copyright 2023, American Chemical Society. c) Accumulative production of ⋅OH monitored by coumarin‐3‐carboxylic acid in the rhizosphere of dried plants and living plants with different amounts of added Fe(II) in the culture solution. Adapted with permission. [36] Copyright 2023, American Chemical Society. d) Diurnal changes of ⋅OH production, DO, redox potential and pH in the rhizosphere under light‐dark cycles. Adapted with permission. [31] Copyright 2022, American Chemical Society. e) Effects of different oxygen availability on the content of $O_{2}^{\cdot -}$ in the rhizosphere of maize. Adapted with permission. [198] Copyright 2024, Elsevier.

O_2_ content variation can directly affect the efficiency of ROS generation and environmental functions by influencing the electron transfer pathway, redox component activity and microbial metabolism. In the intertidal zone, soil O_2_ concentration is regulated mainly by tidal variation and diurnal change (**Table** **4**). Periodic tidal shift can alter the depth of O_2_ penetration at the soil‐water interface, and create unique redox fluctuation conditions for O_2_ activation by electron‐rich material. Zhao et al. showed that when low‐tide soils were exposed to air, a rapid rise in dissolved oxygen (DO) content from <20% air saturation to 100% could trigger rapid single‐electron transfer between Fe(II)/S minerals and O_2_. As shown in **Figure** **8**a, the yield of low‐tide ·OH was elevated by ≈15 nmolg^−1^ at the soil‐water interface with ≈2.5 mm depth compared to the high‐tide period.^[^ ^30^ ^]^ Moreover, Zhao et al. investigated the effect of O_2_ supply mode on ROS production. The results indicated that the cyclic O_2_ supply led to the production of reduced state Fe(II) on the hematite surface under the mediation of tidal variation, and the ·OH yield was enhanced by nearly 4 times (Figure 8b).^[^ ^169^ ^]^ Hence, dynamic O_2_ concentration change can maintain the redox cycle at the active sites on the Fe mineral surface and enhance the ROS yield.

**Table 4:** ROS content from O_2_ activation by electron‐rich material.

Material	Dosage	Reaction time	O_2_ supply	O_2_ content	Main ROS	ROS content	Refs.
Pyrite	40 mg	7 d	Tidal variation	Low tide: 100% air saturation High tide: <20% air saturation	·OH	1.5 µmolL^−1^	[169]
Coastal soil	5 g	1 d	Tidal variation	Low tide: 100% air saturation High tide: <20% air saturation	·OH	≈80 nmol	[30]
Fe minerals	/	3 d	Diurnal change	210 µmolL^−1^	·OH	≈16.8 µmolL^−1^	[31]
Hydrotalcite	/	2.5 d	Diurnal change	6.7 mgL^−1^	·OH	0.8 µmolL^−1^	[36]
Fe minerals	/	24 d	Diurnal change	9.0 m^2^·10^6^·s^−1^	$O_{2}^{\cdot -}$	≈22.4 µmolL^−1^	[198]

“/” represents Not Available.

Aerated tissues and respiratory roots of plants can transport O_2_ from the aboveground part to the roots and interact with soil minerals to generate ROS. As illustrated in Figure 8c, Meng et al. found that the inter‐root DO concentration of normal‐growing rice reached 6.7 mgL^−1^, with an accumulated ·OH of 0.8 µmolL^−1^. However, the DO in the dried rice (with no O_2_ secretion) was only 0.4 mgL^−1^, and almost no ·OH generation.^[^ ^36^ ^]^ Furthermore, significant fluctuation of O_2_ concentration due to light‐dark cycle variation in plant roots can drive ROS production. Dai et al. found that DO concentration increased from 25 µmolL^−1^ (at night) to 210 µmolL^−1^ during the daytime owing to photosynthesis and radial oxygen loss (Figure 8d). Meanwhile, ·OH was generated at a peak rate of 40 nMmin^−1^ in the daytime, and it was much higher than that in the nighttime (<10 nMmin^−1^).^[^ ^31^ ^]^ Therefore, it can be seen that the O_2_ concentration shows a positive correlation with the oxidation reaction activity. Besides, Liu et al. studied the effect of different O_2_ diffusion coefficient (DC_0_) on ROS production. As shown in Figure 8e, with the rise of DC_0_ from 1.47 m^2^·10^6^·s^−1^ to 8.97 m^2^·10^6^·s^−1^, the $O_{2}^{\cdot -}$ production increased by ≈16 µmol·L^−1^·kg^−1^.^[^ ^30^ ^]^ Consequently, an appropriate rise of DC_0_ can promote ROS generation. Nevertheless, too high DC_0_ may cause passivation of the mineral surface, hinder electron transfer, and increase the economic cost.

In summary, O_2_ content has a positive correlation with ROS production. Both the rapid increase in DO during tidal low tide and the photosynthesis‐driven increase in inter‐root O_2_ concentration during daytime can enhance ROS production. Therefore, in situ remediation of polluted sites can be conducted through the selection of plants with high O_2_ secretion from the root system or the increase of O_2_ in soil pore space via soil loosening.

### 5.3 Soil Organic Matters

**Figure 9:**
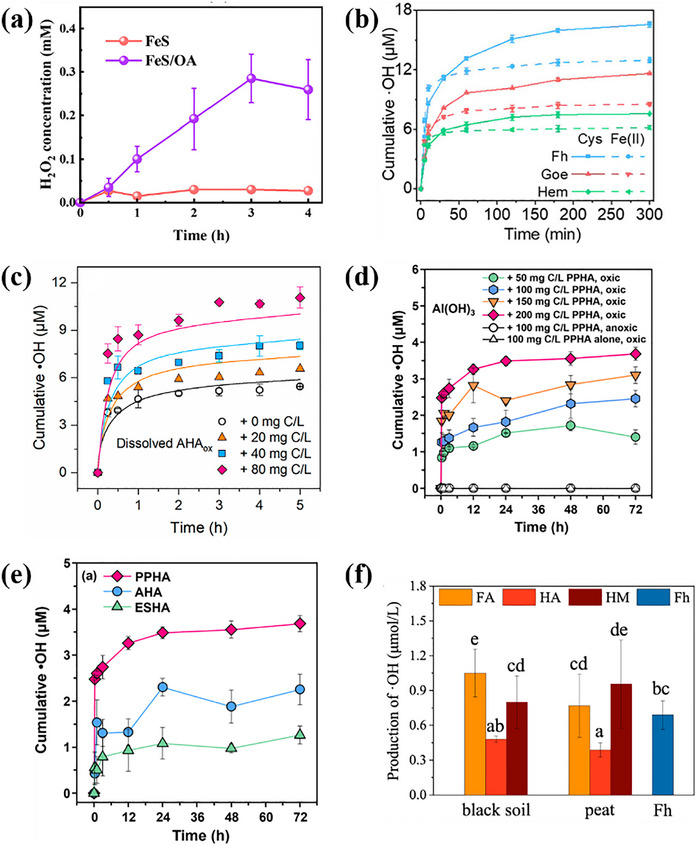
a) H_2_O_2_ production in pure FeS and FeS/OA systems. Adapted with permission. [181] Copyright 2023, American Chemical Society. b) Cumulative accumulation of ·OH in Fe oxyhydroxide–Cys systems or Fe oxyhydroxide–Fe(II) systems under oxic conditions. Adapted with permission. [71] Copyright 2024, American Chemical Society. c) Effect of exogenous dissolved AHA_ox_ on ⋅OH production from AHA_red_ suspension oxygenation. Adapted with permission. [32] Copyright 2022, American Chemical Society. d) Influence of initial concentration of PPHA on ·OH production. e) Influence of HA types on ·OH production. Adapted with permission. [182] Copyright 2025, Elsevier. f) Production ·OH in both SOM‐free and SOM‐containing systems. Adapted with permission. [175] Copyright 2022, American Chemical Society.

The redox state, component composition and content of soil organic matters, as well as the interaction between soil organic matter and minerals, can affect the ROS yield (**Table** **5**). As shown in **Figure** **9**a, the single FeS system produced only 0.02 mM of H_2_O_2_ yield from O_2_ activation in 3 h. The addition of OA increased the H_2_O_2_ output to 0.3 mM.^[^ ^181^ ^]^ Moreover, compared with the single hydrotalcite system, the addition of Cys improved the ⋅OH production from 13 to 16.5 µmolL^−1^ in 5 h (Figure 9b).^[^ ^71^ ^]^ Therefore, the addition of organic matter can broaden the pathway of O_2_ activation and boost the ROS yield. The effect of different AHA concentrations in sediments on ⋅OH production over 5 h was investigated by Yu et al. As shown in Figure 9c, the ⋅OH yield rose from 5 to 11 µM as the AHA concentration increased from 0 to 80 mg CL^−1^.^[^ ^32^ ^]^ Besides, the ⋅OH yield within 72 h was elevated by 2.3 µM as the PPHA concentration in clay minerals grew from 50 to 200 mg C/L by Pu et al. (Figure 9d).^[^ ^182^ ^]^ Hence, the organic matter concentration is positively correlated with ROS yield in the low concentration range (<200 mg CL^−1^). If the concentration is too high beyond the capacity of the mineral adsorption sites, the ROS output may plateau or decline.

**Table 5:** ROS content from O_2_ activation by Organic matter.

Organic matter	Dosage	Reaction time	Main ROS	ROS content	Refs.
HA	36.5 mg Cg^−1^	24 h	·OH	117.4 µmolm^−2^	[30]
Cys	20 mM	5 h	·OH	16.6 µM	[71]
HM	25 gL^−1^	168 h	$O_{2}^{\cdot -}$	/	[180]
HA	0.8 g CL^−1^	5 h	·OH	7.8 µM	[32]
FA	100 mg CL^−1^	5 h	·OH	1.05 µM	[175]
HA	100 mg CL^−1^	5 h	·OH	0.46 µM	[175]
HM	100 mg CL^−1^	5 h	·OH	0.79 µM	[175]
OA	1.5 mM	4 h	·OH	128 µM	[181]
OA	1.5 mM	3 h	H_2_O_2_	290 µM	[181]
PPHA	200 mg CL^−1^	72 h	·OH	3.7 µM	[182]
AHA	200 mg CL^−1^	72 h	·OH	2.3 µM	[182]
ESHA	200 mg CL^−1^	72 h	·OH	1.3 µM	[182]

Cys: Cysteine; OA: Oxalic acid; FA: Fulvic acid; Humic acid; HM: Humin; PPHA: Pahokee peat humic acid; AHA: Aldrich humic acid; ESHA: Elliott soil humic acid. “/” represents Not Available.

The influence of soil organic matters on ROS production varies with the changes of its fractions. The impact of Pahokee peat humic acid (PPHA), Aldrich humic acid (AHA), and Elliott soil humic acid (ESHA) on ROS generation was studied by Pu et al. As indicated in Figure 9e, ⋅OH production over 72 h was ranked as PPHA (3.7 µM) > AHA (2.3 µM) > ESHA (1.3 µM).^[^ ^182^ ^]^ This sequence is consistent with the order of three HA electron‐delivery capacity (EDC), and indicates that the large EDC of HA is favorable to the O_2_ activation for generating ⋅OH. Furthermore, Han et al. demonstrated that compared to the single hydromorphite system, the addition of solid FA and HM extracted from black soil increased the ⋅OH yield within 5 h by 0.4 µmolL^−1^ and 0.2 µmolL^−1^, respectively. However, the addition of solid HA decreased the ⋅OH yield by 0.22 µmolL^−1^ (Figure 9f).^[^ ^175^ ^]^ It is attributable to the higher molecular weight and high EDC of HA than FA and HM, so as to consume ⋅OH. Therefore, FA‐rich organic materials such as compost fulvic acid can be preferred for the remediation of contaminated soils. If it is needed to retain organic carbon in soil, a high proportion of solid HA like rotted cow dung can be used to amend the soil. Meanwhile, the mineral surface covered by solid‐state HA may hinder direct contact between Fe(II) and O_2_ and inhibit electron transfer compared to dissolved‐state PPHA, AHA, and ESHA described in the previous example. On the other hand, dissolved‐state HA can form soluble complexes with Fe(II) and promote the homogeneous Fenton reaction. Hence, the phase state of HA plays a decisive role in O_2_ activation.

### 5.4 Sunlight Intensity

**Figure 10:**
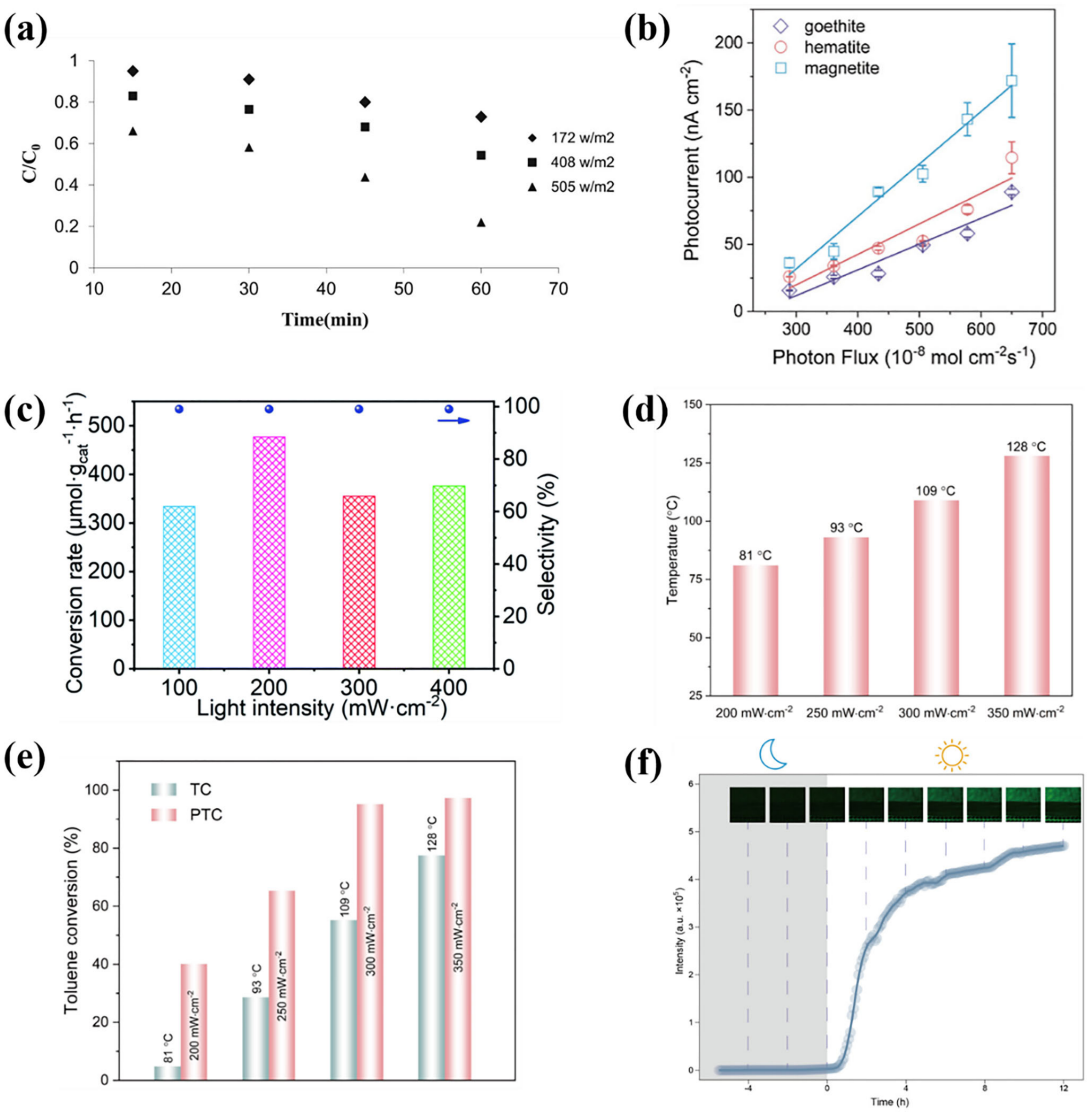
a) Effect of UV intensity on the removal efficiency of pollutants (initial 2, 4‐D concentration = 25 mgL^−1^, pH = 7, cross‐section = 200 cm^2^, UV intensity = 172, 408 and 505 Wm^−2^, nanoparticle suspension concentration 3% and 1% WO_3_ doped ZnO). Adapted with permission. [200] Copyright 2022, Taylor and Francis Ltd. b) Photocurrent production at different photon fluxes. Adapted with permission. [201] Copyright 2022, American Chemical Society. c) The results from the Pd/BiOBr photocatalyst under the UV‐visible irradiation of different optical power densities. Adapted with permission. [202] Copyright 2020, Royal Society of Chemistry. d) The surface equilibrium temperature of 0.5 Cu‐MnO_2_ under different light intensity. e) The photothermal catalytic (PTC) performance of 0.5 Cu‐MnO_2_ under different light intensity and thermocatalytic (TC) performance at the corresponding equilibrium temperature. Adapted with permission. [107] Copyright 2024, Elsevier. f) Time series of ROS production under dark and light conditions. Adapted with permission. [31] Copyright 2022, American Chemical Society.

Light intensity can directly regulate the number of photo‐generated carriers, and indirectly affect thermal effect and biological process; consequently, influences the O_2_ activation efficiency (**Table** **6**). A moderate enhancement of light intensity can increase the number of photons absorbed per unit time, produce more photoelectrons to promote O_2_ activation, and generate more ROS. The number of photons absorbed per unit time can increase via a moderate rise in light intensity, so more photoelectrons are generated to boost O_2_ activation and generate more ROS. Bhatia et al. studied the effect of light intensity on ATL degradation by TiO_2_‐G. The results showed that with light intensity increase from 250 to 1000 Wm^−2^, the ATL removal rate rose from 65% to 95%.^[^ ^199^ ^]^ This was due to an increase in the^−^ under high light intensity, accelerated the conversion from O_2_ to $O_{2}^{\cdot -}$. As shown in **Figure** **10**a, Zandsalimi et al. found that as the light intensity increased from 172 to 505 Wm^−2^, the removal rate of 2,4‐dechlorophenoxyacetic acid with WO_3_/ZnO improved from 27% to 78%.^[^ ^200^ ^]^ Moreover, Zheng et al. exposed Fe minerals to different intensity of simulated sunlight. The results indicated that the photocurrent of Fe minerals was linearly related to incident light intensity (Figure 10b).^[^ ^201^ ^]^ It was found that an appropriate increase in light intensity could improve the generation efficiency of photogenerated carriers from Fe minerals. Therefore, the linear response characteristic of light intensity in Fe‐rich soil can be utilized to generate ROS in situ through solar energy, and reduce remediation costs. Furthermore, for materials such as TiO_2_‐G, WO_3_/ZnO, and Fe minerals, maximum ROS production can be achieved by full utilization of the midday peak sunlight period for surface soil remediation. However, when the light intensity exceeds the specific threshold of the electron‐rich material, it will inhibit O_2_ activation. As depicted in Figure 10c, Li et al. demonstrated that the conversion rate of toluene via Pd/BiOBr increased by 143.0 µmol·g^−1^·h^−1^ under light intensity ranging from 100 to 200 mWm^−2^. Nevertheless, with further enhancement of light intensity to 300 mWm^−2^, the conversion rate of toluene decreased by 119.6 µmol·g^−1^·h^−1^.^[^ ^202^ ^]^ Excessively high light intensity causes the plasma effect of Pd to trigger a reverse electron flow, reduce the electron density at Pd sites, and weaken O_2_ activation capacity. Therefore, in order to achieve efficient and energy‐saving surface soil remediation, light intensity should be precisely controlled according to material characteristics, and shade regulation should be used if necessary.

**Table 6:** The degradation of pollutants via O_2_ activation by electron‐rich materials under different light intensity.

Material	Dosage	Reaction time	Light source	Light intensity [Wcm^−2^]	Main ROS	Pollutant	Initial concentration	Efficiency	Ref.
TiO_2_‐G	1.5 gL^−1^	1 h	Xe lamp	250	·OH	Atenolol	25 mgL^−1^	11.9 µmol·g^−1^·h^−1^	[199]
				550				40.1 µmol·g^−1^·h^−1^	[199]
				750				45.1 µmol·g^−1^·h^−1^	[199]
				1000				55.1 µmol·g^−1^·h^−1^	[199]
WO_3_/ZnO	0.04 gL^−1^	1 h	UV lamp	172	·OH	2,4‐D	25 mgL^−1^	762.8 µmol·g^−1^·h^−1^	[200]
			Xe lamp	408				1327.8 µmol·g^−1^·h^−1^	[200]
			Xe lamp	505				2203.5µmol·g^−1^·h^−1^	[200]
Pd/BiOBr	2.5 gL^−1^	5 h	Xe lamp	100	$O_{2}^{\cdot -}$	Toluene	2.9 mmolL^−1^	333.8 µmol·g^−1^·h^−1^	[202]
				0.2				476.8 µmol·g^−1^·h^−1^	[202]
				0.3				357.2µmol·g^−1^·h^−1^	[202]
				0.4				377.3 µmol·g^−1^·h^−1^	[202]
Cu‐MnO_2_	100 mg	0.5 h	Xe lamp	0.1	O^−^	Toluene	300 ppm	40.20%	[107]
				0.2				64.80%	[107]
				0.3				95.70%	[107]
				0.4				97.10%	[107]

G: Graphene; 2,4‐D: 2,4‐dechlorophenoxyacetic acid.

Light intensity can indirectly regulate O_2_ activation through thermal effect. Wang et al. found that high light intensity can cause localized heating of Cu‐MnO_2_, promote O_2_ adsorption, and activate it into O^−^. The data showed that as the light intensity increased from 200 to 300 mW/cm^2^, the surface temperature of Cu‐MnO_2_ rose by 28 °C, and the degradation rate of toluene increased by 45.5% (Figure 10d,e).^[^ ^107^ ^]^ The highly efficient activation process of O_2_ is primarily driven by light, while thermal effects dominate lattice oxygen migration and accelerate intermediate conversion. In combination, it achieves the dynamic cycle of active sites. Dynamic changes in light intensity indirectly affect O_2_ activation by regulation plant photosynthesis. As demonstrated in Figure 10f, it was observed that ROS production was minimal under dark conditions, while light exposure rapidly triggered a significant increase in ROS generation. Higher light intensity can enhance photosynthesis, and indirectly promote O_2_ diffusion to the rhizosphere for ROS activation.^[^ ^31^ ^]^ Hence, for mid‐layer soil with insufficient light, ROS production can be indirectly promoted via thermal effect or oxygen supply from plant roots.

## 6 Conclusion and Perspective

As cheap and accessible oxidant precursors, O_2_ activation by electron‐rich materials has gained much attentions in recent years. In this paper, the activation mechanisms of O_2_ to produce ROS by different electron‐rich materials and their applications in soil remediation were comprehensively summarized, and the effects of environmental factors, such as pH, O_2_ content, organic matter, and sunlight intensity, on O_2_ activation were investigated. The main conclusions included:

1. Different electron‐rich materials have been used to construct multidimensional O_2_ activation networks by the design of electron transfer pathways. In contrast to traditional AOPs, the source of oxidants has shifted from external addition to in situ utilization. The reaction pathways have expanded from a single radical‐dominated process to a multi‐pathway system combining radical and non‐radical mechanisms. In terms of energy utilization, a transition has been achieved from reliance on artificial energy sources to coupling with light energy, mechanical energy, and geochemical energy. Fe‐based materials can activate O_2_ to generate $O_{2}^{\cdot -}$, H_2_O_2_, and ·OH via Fe(II)/Fe(III) cycles, surface defects, and ligand synergistic pathways. Bi‐based semiconductor heterojunctions and piezoelectric materials can achieve breakthroughs in the O_2_ spin‐forbidden barrier through photo or mechanical energy‐driven electron transfer. An efficient O_2_ activation can be realized by dynamic Cu(I)/Cu(II) cycle of Cu‐based materials. Carbon‐based materials promote O^*^ generation by the weakness of O─O bonds through porous domain restriction, N doping, and edge defects.
2. For Fe minerals, ·OH yields can be significantly enhanced by exposure of highly active crystal planes, reduction of crystallinity, and introduction of redox fluctuations. Moreover, the synergistic effects of metal doping, ligand incorporation, and plant roots can promote the O_2_ activation from Fe minerals and improve the efficiency of soil pollution remediation. On the other hand, the reduced state HM can provide electrons directly via phenolic hydroxyl groups and EPFR. While oxidized state HM can form a more stable electron transfer network through the structural reconfiguration induced by the mineral interface.
3. pH can determine the O_2_ activation pathway. Acidic (pH 5‐6) is dominated by homogeneous Fenton. Neutral to weakly alkaline environments (pH 7–8) rely on heterogeneous interfacial reactions, and strongly alkaline environments (pH>8) can weaken the negative charge effect through ligand complexation. Moreover, low tide periods as well as plant photosynthesis during daytime can increase O_2_ content in soil and promote ROS production. Soil organic matter concentration is positively correlated with ROS production at less than 200 mg CL^−1^. Furthermore, light intensity needs to be adjusted according to the characteristics of different electron‐rich materials. Excessive light intensity can reduce O_2_ activation efficiency.

Although some progress has been made to investigate the O_2_ activation in soil by electron‐rich materials, there remain challenges to be overcome in future research for its engineering application in soil remediation:

1. Given that Bi‐based and Cu‐based materials have not been directly applied in soil remediation, materials with adjustable pore channels or carrier composite structures should be designed to enhance mass transfer between O_2_ and pollutants. The efficiency of electron migration within the solid phase should be improved through conductive networks, heterojunctions, or electron relays. Pathways such as visible light response enhancement, bioluminescence coupling or electrically assisted excitation should be explored to overcome light attenuation at the soil surface. Concurrently, the stability and ecological safety of materials in soil should be evaluated. Besides, the activation mechanisms of O_2_ by electron‐rich materials is mainly investigated in a single material system. Considering the complex soil matrix, the multicomponent synergistic relationships between minerals, organic matter, and microorganisms are still unclear. The coupling of in situ characterization techniques with DFT and molecular dynamics calculations can be applied to explore the laws of electron‐leaping pathways and active sites in composites. Particular attentions need to be paid to the charge transfer resistance at heterogeneous interfaces in soil. To enhance the long‐term stability of electron‐rich materials, active sites can be protected by the construction of core‐shell structures, and in situ regeneration of active sites may be achieved through metal valence state cycle. The passivation process may be slowed by regulation of the dynamic exposure of surface defects via vacancy engineering or the introduction of surface ligands or functional groups. Moreover, extensive research focuses on enhancing O_2_ activation capacity through material modification, but most works remain confined to mechanism verification. Subsequent studies should apply the modified materials to real aquatic or soil systems to investigate the effects of coexisting ions, organic matter, minerals, and other factors on pollutant removal performance.
2. Most of the existing researches focus on single pollutants, while the real soil often faces the compound pollution including heavy metals, organic pollutants, and even extreme environmental stresses such as high salt, strong acid, and strong alkali. To develop multifunctional composite systems, the construction of multifunctional composites, introduction of multiphase interface electronic pathways, and regulation of ROS species ratios are necessary. Concurrently, a low‐energy dynamic remediation process should be designed based on the natural characteristics of soil redox fluctuations. Furthermore, microbial metabolism and activation of O_2_ by electron‐rich materials can be integrated in the subsequent research, and it is expected to realize the synergistic effect of soil pollutant elimination and ecological function recovery. It is necessary to optimize O_2_ mass transfer and electron migration pathways to promote the large‐scale application of O_2_ activation. Plants with strong radial oxygen loss and micro/nano‐bubble aeration should be selected to ensure a sustainable supply of O_2_. Moreover, pilot‐scale and in situ remediation experiments should be conducted to evaluate the stability of the reaction under different soil structures, moisture contents, and O_2_ diffusion conditions.
3. The long‐term application of electron‐rich materials may change the structure of soil microbial communities and biogeochemical cycles (e.g., carbon, nitrogen, sulfur cycles). The intergenerational impacts on soil health should be assessed by metagenomics and metabolomics pathways. Besides, a green remediation strategy based on life cycle assessment should be constructed in the future, and natural minerals (e.g., GR and sulfurous Fe ore) or waste biomass materials should be prioritized to reduce the risk of secondary pollution. In addition, the O_2_ activation parameters should be dynamically regulated by the combination of intelligent sensing and machine learning to realize precise remediation and efficient resource utilization.

## Conflict of Interest

The authors declare no conflict of interest.
